# Crimean Congo hemorrhagic fever virus nucleoprotein and GP38 subunit vaccine combination prevents morbidity in mice

**DOI:** 10.1038/s41541-024-00931-y

**Published:** 2024-08-14

**Authors:** Elif Karaaslan, Teresa E. Sorvillo, Florine E. M. Scholte, Troy Justin O’Neal, Stephen R. Welch, Katherine A. Davies, JoAnn D. Coleman-McCray, Jessica R. Harmon, Jana M. Ritter, Scott D. Pegan, Joel M. Montgomery, Jessica R. Spengler, Christina F. Spiropoulou, Éric Bergeron

**Affiliations:** 1 Viral Special Pathogens Branch, Division of High-Consequence Pathogens and Pathology, National Center for Emerging and Zoonotic Infectious Diseases, Centers for Disease Control and Prevention, Atlanta, GA USA; 2 Division of Biomedical Sciences, University of California Riverside, Riverside, CA USA; 3 U.S. Department of Agriculture, Agricultural Research Service, Zoonotic and Emerging Disease Research Unit, National Bio and Agro-Defense Facility, Manhattan, KS USA; 4 Infectious Disease Pathology Branch, Division of High Consequence Pathogens and Pathology, Centers for Disease Control and Prevention, Atlanta, GA USA; 5 Department of Pharmaceutical and Biomedical Sciences, College of Pharmacy, University of Georgia, Athens, GA USA

**Keywords:** Protein vaccines, Viral infection, Virology, Antibodies

## Abstract

Immunizing mice with Crimean-Congo hemorrhagic fever virus (CCHFV) nucleoprotein (NP), glycoprotein precursor (GPC), or with the GP38 domain of GPC, can be protective when the proteins are delivered with viral vectors or as a DNA or RNA vaccine. Subunit vaccines are a safe and cost-effective alternative to some vaccine platforms, but Gc and Gn glycoprotein subunit vaccines for CCHFV fail to protect despite eliciting high levels of neutralizing antibodies. Here, we investigated humoral and cellular immune responses and the protective efficacy of recombinant NP, GP38, and GP38 forms (GP85 and GP160) associated with the highly glycosylated mucin-like (MLD) domain, as well as the NP + GP38 combination. Vaccination with GP160, GP85, or GP38 did not confer protection, and vaccination with the MLD-associated GP38 forms blunted the humoral immune responses to GP38, worsened clinical chemistry, and increased viral RNA in the blood compared to the GP38 vaccination. In contrast, NP vaccination conferred 100% protection from lethal outcome and was associated with mild clinical disease, while the NP + GP38 combination conferred even more robust protection by reducing morbidity compared to mice receiving NP alone. Thus, recombinant CCHFV NP alone is a promising vaccine candidate conferring 100% survival against heterologous challenge. Moreover, incorporation of GP38 should be considered as it further enhances subunit vaccine efficacy by reducing morbidity in surviving animals.

## Introduction

Crimean-Congo hemorrhagic fever virus (CCHFV) is a tick-borne virus with a wide geographical distribution due to the broad range of ixodid ticks, CCHFV’s reservoir and vector^1,2^. In humans, the resulting disease, Crimean-Congo hemorrhagic fever (CCHF), manifests with a sudden onset of nonspecific flu-like symptoms; hemorrhage and neurological symptoms may follow. Disseminated intravascular coagulation, multiple organ failure, and shock ensue in more severe cases^3,4^. Infection can be fatal, with mortality rates up to 40%^5^. CCHFV is endemic in Africa, the Balkans, the Middle East, and Asia; it is considered an emerging/re-emerging pathogen due to new incidents from previously unreported geographical locations as well as outbreaks in endemic regions^6–10^. CCHFV is included in the top-priority pathogens list of the World Health Organization, and currently, no approved vaccine or treatment is available^11^.

CCHFV belongs to the *Orthonairovirus* genus, family *Nairoviridae*. Like other orthonairoviruses, CCHFV is a negative-stranded RNA virus characterized by a tripartite genome. The small (S) segment encodes nucleoprotein (NP), and a nonstructural S protein (NSs) is encoded in opposite orientation. The medium (M) and large (L) segments encode glycoprotein precursor (GPC) and L protein, respectively^12^. GPC undergoes glycosylation and several cleavage events mediated by host proteases residing in the secretory pathway^12^, generating surface glycoproteins Gn and Gc; secreted proteins GP160, GP85, and GP38; non-structural M protein (NSm); and mucin-like domain (MLD)^13–17^. GP38 represents the furin-cleaved form of GP160 or GP85^14^, while GP85 and 160 are MLD-linked forms of GP38 that differ in O-glycan content^18^. The L protein contains de-ubiquitination and de-ISGylation activities, along with RNA-dependent RNA polymerase critical for genome replication and transcription^19–21^.

Traditionally, viral surface glycoproteins are pivotal targets for vaccine development due to their capacity to stimulate the production of neutralizing antibodies and their potential to confer sterilizing immunity^22–25^. However, for CCHFV, the development of neutralizing antibodies is not always correlated with protection^26^, and protection conferred by a DNA vaccine utilizing surface glycoproteins as antigens does not depend on neutralizing antibodies in IFNAR and immunosuppressed mice^27^. CCHFV NP is essential, functioning in ribonucleoprotein particle formation and possibly in budding^28^ and immune evasion^29,30^, and is abundant in infected cells. It is highly immunogenic^31–34^ and highly conserved among phylogenetic clades^31,32,35,36^, properties that make NP an attractive vaccine antigen. GP38 is required for proper processing and trafficking of Gn and Gc precursors^13,37^ and is immunorelevant in mice and humans^38,39^. Additionally, NP and GP38 (for which antibody responses are non-neutralizing) confer protection when used as vaccine antigens^40–44^, and non-neutralizing antibodies targeting GP38 demonstrate therapeutic efficacy when mice are treated before or after infection^45–47^.

Subunit vaccines present numerous advantages over some vaccine platforms. By definition, they include no live or inactivated pathogens, rendering them inherently safe, with lower reactogenicity and production costs^48,49^. Moreover, these vaccines offer significant flexibility in selecting, modifying, and combining antigens, allowing tailored approaches to vaccination^49,50^. Despite potential concerns regarding suboptimal immunogenicity, recombinant protein subunit vaccines have established themselves as excellent vaccine platforms, protecting against human papillomavirus^51^, hepatitis B virus^52^, influenza virus^53^, and SARS-CoV-2, among others^54^. The potential of subunit vaccines against CCHFV has not yet been explored extensively. Only one study investigated the protective efficacy of glycoproteins Gn and Gc, finding that vaccination induced neutralizing antibodies but failed to confer protection^26^.

In this study, we explored the protective efficacy of recombinant NP and GP38 individually and in combination. We also investigated the effect of MLD on immune responses by vaccinating animals with MLD-linked precursor forms of GP38: GP85 or GP160. Our findings revealed that the presence of MLD on GP85 and GP160 influenced the robustness and characteristics of anti-GP38 immune responses. Vaccination with GP38 and other GP38-containing proteins did not result in significant protection following challenge with a heterologous strain of CCHFV, and presence of the MLD was associated with worsened clinical chemistry and increased viral RNA (vRNA) in the blood. In contrast, recombinant NP elicited potent immune responses that conferred complete protection from lethal outcome following heterologous challenge. Furthermore, vaccination with the NP+GP38 combination enhanced protection compared to NP alone by reducing clinical signs of disease in survivors. These results underscore the potential of recombinant NP+GP38 as a promising vaccine candidate for mitigating CCHF.

## Results

### Recombinant NP and GP160, GP85, and GP38 immune responses in immunocompetent mice

**Fig. 1:**
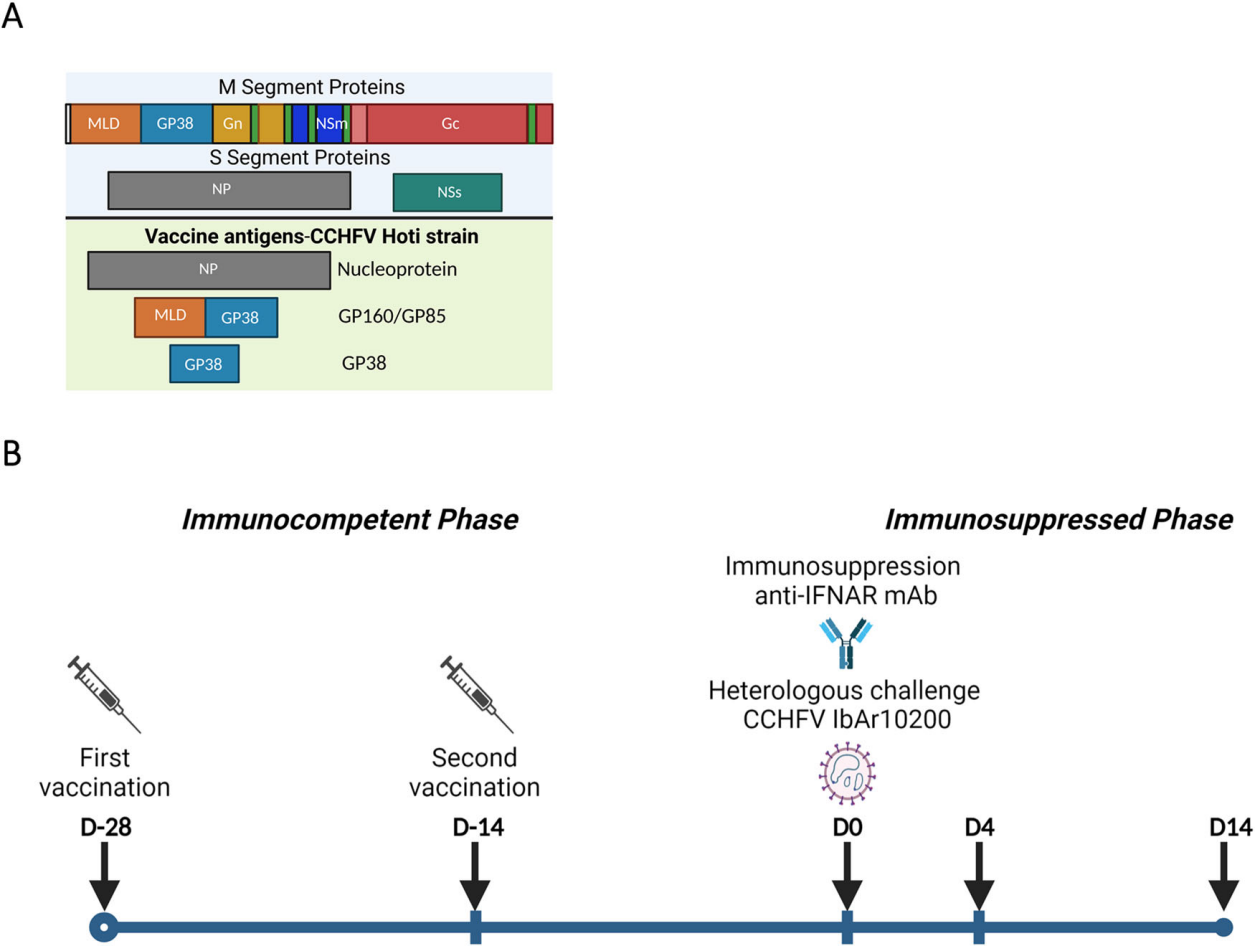
Vaccine antigens and study timeline. **A** Schematic representation of CCHFV proteins encoded from S and M segments. Transmembrane domains indicated in green; signal peptide indicated in white. The second half of the figure shows the CCHFV proteins used in this study as vaccine antigens. Protein sequences were derived from CCHFV Hoti strain (accession numbers: S segment DQ133507.1; M segment EU037902.1). **B** Timeline showing vaccination, immunosuppression, and challenge schedule. Groups of 6 mice were vaccinated subcutaneously with NP + GP38, NP, GP38, GP85, or GP160. For pre-challenge studies, groups of mice were euthanized 14 days after their vaccination (on D14). Another group received two vaccinations with a two-week interval and was euthanized 14 days after the second vaccination (on D0). For post-challenge studies, animals received two vaccinations two weeks apart. Fourteen days after their second vaccination, animals were immunosuppressed with anti-interferon alpha/beta receptor subunit 1 monoclonal antibody (anti-IFNAR-1 mAb) MAR1-5A3 intraperitoneally before being subcutaneously challenged with 1000 TCID_50_ of recombinant CCHFV IbAr10200 on D0. One group of mice was euthanized on D4 to evaluate immune responses, disease progression, and virus spread. Another group was monitored for 14 days to evaluate protective efficacy of the vaccines; survivors were euthanized on D14. Graphical illustrations were prepared with BioRender (www.biorender.com).

The native monomeric form of CCHFV Hoti strain NP was expressed in bacteria, CCHFV Hoti GP160, GP85, and GP38 were expressed in a mammalian expression system, and proteins were purified by affinity and size exclusion chromatography (Supplementary Fig. 1A–D)^55,56^. Monophosphoryl lipid A, a TLR4 agonist, and AddaVax, a squalene-based oil-in-water nano-emulsion, were selected as adjuvants based on a previously published study^44^. Groups of immunocompetent C57BL/6J mice (n = 6/group) were vaccinated subcutaneously (SC) with NP, GP38, GP85, or GP160, or with the combination NP+GP38 (Fig. 1A). A group of animals was euthanized 14 days after the first vaccination, and a second group was euthanized 14 days after the second vaccination (Fig. 1B). The differences in antibody titers, subclasses, and avidity after first and second vaccinations were investigated.

**Fig. 2:**
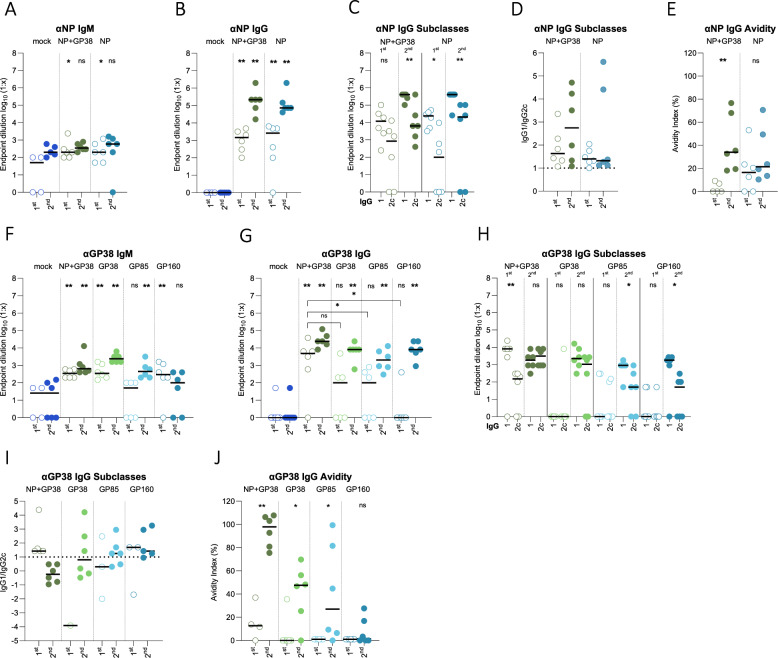
Humoral immune responses after the first and second vaccinations. NP- and GP38-specific IgM (**A**, **F**) and IgG (**B**, **G**) responses after the first and second vaccinations were determined as endpoint titers in plasma samples. Anti-NP **C** and anti-GP38 **H** IgG1 and IgG2c titers were determined as endpoint titers in plasma samples. The IgG1 to IgG2c ratios were determined by dividing the endpoint titers of IgG1 to IgG2 of individual animals and presented in **D** for anti-NP antibodies and in **I** for anti-GP38 antibodies. The dotted horizontal line represents IgG1 to IgG2c ratio = 1. The avidity indices of anti-NP **E** and anti-GP38 **J** antibodies from vaccinated animals after the first and second vaccinations were determined. For the avidity of IgG antibodies, areas under the curves (AUC) of 7-point dilutions of urea-treated and untreated samples were determined and the avidity index calculated as follows: (AUC of the urea-treated sample/AUC of untreated sample) × 100. Each dot represents the mean value of the replicates from each animal and the horizontal line represents the median value for the group. All samples were tested in duplicate. Two-tailed nonparametric *t* tests and ordinary one-way ANOVA (*p < 0.05; **p < 0.001; ***p < 0.0003) were used for statistical analyses when applicable. Statistical analyses compared IgM and IgG titers from each vaccine group with those of the mock-vaccinated animals at corresponding timepoints. For IgG subclasses, statistical analyses compared IgG1 and IgG2 titers of individual vaccine groups after the first and second vaccination. For IgG avidity determination, statistical analyses were performed with IgG titers after first and second vaccinations.

Similar levels of total anti-NP IgM and IgG were detected in animals vaccinated with NP alone and with NP+GP38 following the first and second vaccinations (Fig. 2A, B). Ratios of IgG1 over IgG2c titers >1 were detected in these groups, indicating a Th2-biased immune response (Fig. 2C, D). Following the second dose, both vaccinations induced antibodies with similar avidity (Fig. 2E).

Moderate levels of anti-GP38 IgM and IgG antibodies were detected in NP+GP38, GP38, GP85, and GP160 groups after the second vaccination (Fig. 2F, G). After the second vaccination, the subclasses of these IgG antibodies included more IgG1 in GP85- and GP160-vaccinated animals, while the GP38-vaccinated group developed a more balanced response, with a ratio close to 1, and NP+GP38 induced IgG1 to IgG2c ratios <1 (Fig. 2H, I). Even though all groups had similar IgG titers, the avidity of these antibodies differed, with the highest anti-GP38 IgG avidity detected in the NP+GP38 and GP38 groups (Fig. 2J).

**Fig. 3:**
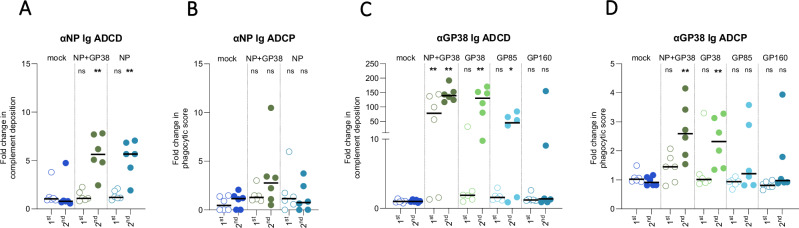
Fc-mediated functions of anti-NP and anti-GP38 antibodies following vaccination. Fc-mediated functions of anti-NP and anti-GP38 antibodies were investigated after first and second vaccinations. ADCD function of anti-NP **A** and anti-GP38 **C** antibodies are represented as the fold change in complement deposition over mock-vaccinated group. ADCP function of anti-NP **B** and anti-GP38 **D** antibodies represented as fold change in phagocytic score over mock-vaccinated animals. Each dot represents the mean value of the replicates from each animal and the horizontal line represents the median. Two-tailed nonparametric *t* tests and ordinary one-way ANOVA (*p < 0.05; **p < 0.001; ***p < 0.0003) were used for statistical analyses when applicable. Statistical analyses compare vaccine groups and mock-vaccinated animals at corresponding timepoints.

We then determined antibody-dependent complement deposition (ADCD) and antibody-dependent cellular phagocytosis (ADCP) of the anti-NP and anti-GP38. We detected ADCD function in anti-NP antibodies after the second vaccination in both NP+GP38- and NP-vaccinated animals, and these antibodies did not exhibit ADCP (Fig. 3A, B). On the other hand, anti-GP38 antibodies displayed both ADCD and ADCP, depending on the vaccination group (Fig. 3C, D). Following the second vaccination, similar activity was detected in NP+GP38- and GP38-vaccinated animals; less ADCD was seen in the GP85 group, while only two GP160-vaccinated animals had detectable ADCD (Fig. 3C). Similar levels of ADCP were observed in the NP+GP38- and GP38-vaccinated groups, while 4 of 6 GP85- and GP160-vaccinated mice showed no detectable ADCP (Fig. 3D).

**Fig. 4:**
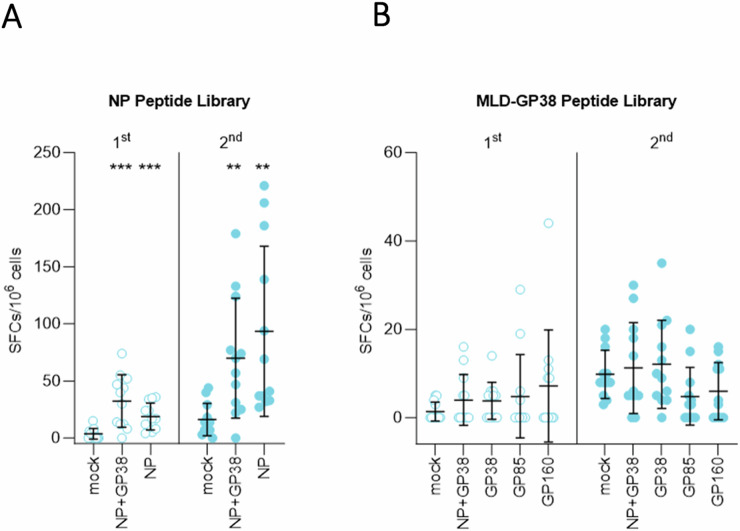
Cellular immune responses after vaccination. Cellular immune responses were investigated using peptide libraries of CCHFV 10200 NP and Hoti MLD-GP38. IFN-gamma recall responses detected from splenocytes of mice to **A** NP and **B** MLD-GP38 peptide libraries. In each graph, the left panel shows responses following the first vaccination, and the right panel shows responses following the second vaccination; data are represented as number of spot-forming cells (SFCs) in 10^6^ peptide-stimulated splenocytes. All samples were tested in duplicate, and results were normalized to PMA control of each sample. Horizontal line represents the median value for the group. Two-tailed nonparametric *t* tests and ordinary one-way ANOVA (**p < 0.001; ***p < 0.0003) were used for statistical analyses when applicable. Statistical analyses compare vaccine groups and mock-vaccinated animals at corresponding timepoints.

Next, we evaluated the induction of cellular immune responses by determining the number of IFN-gamma-producing T cells in splenocytes using peptide libraries spanning the entire NP and a library spanning the MLD-GP38. NP- and NP+GP38-vaccinated animals developed significant responses to the NP peptide library (Fig. 4A). On the other hand, no MLD-GP38-specific IFN-gamma-producing T cells were detected in NP+GP38-, GP160-, GP85-, or GP38-vaccinated groups following the first and second vaccinations (Fig. 4B).

### CCHFV challenge of vaccinated mice

**Fig. 5:**
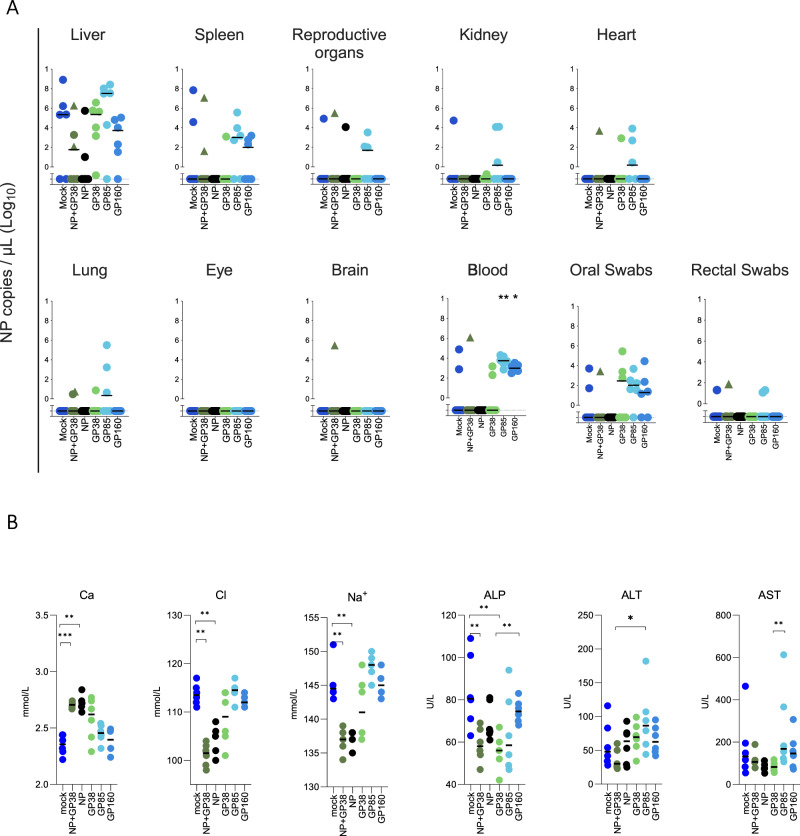
Viral RNA levels and clinical chemistry of vaccinated animals 4 days after challenge with lethal dose of CCHFV. After second vaccination, animals were immunosuppressed and challenged subcutaneously with a lethal dose of CCHFV IbAr10200. Four days after challenge, 6 animals from each vaccine group were euthanized and viral RNA levels and clinical chemistry parameters were analyzed. **A** Viral RNA levels in liver, spleen, reproductive organs, kidney, heart, lung, eye, brain, blood, and oral and rectal swabs were determined. Closed triangles represent the two animals in this group that showed liver and spleen pathology similar to mock- vaccinated animals. **B** Levels of calcium (Ca), chloride (Cl), sodium (Na^+^), alkaline phosphatase (ALP), alanine transaminase (ALT), and aspartate aminotransferase (AST) were determined from whole blood within 1 h of collection and represented as mg/dL (CA), mmol/L (Cl, Na^+^) or U/L (ALP, ALT, AST). Horizontal line represents the median value for the group. Multiple comparisons were performed using a two-way ANOVA. *p* values were adjusted for multiple comparisons using the two-stage linear set-up procedure of Benjamini, Krieger, and Yekutieli (*p < 0.05; **p < 0.001; ***p < 0.0003).

We next investigated the protective efficacy of each vaccine antigen (NP, NP+GP38, GP38, GP85, and GP160). Since CCHFV infection is not lethal in immunocompetent mice, we suppressed the innate immune response by treating mice with monoclonal antibody (mAb) mAb-5A3, an anti-interferon receptor-1 mAb that neutralizes type-I IFN signaling^27,38,57^. Fourteen days after the second vaccination, animals were given mAb-5A3 and immediately infected subcutaneously (SC) with a lethal dose of recombinant CCHFV IbAr10200, a strain heterologous to the antigens (which were strain Hoti). Animals were monitored daily for clinical signs and weight loss, and subgroups of mice were euthanized 4 days after challenge to examine early viral dissemination and immune responses (Fig. 1B). On day 4, disease had not progressed enough to discern subtle differences between groups; however, analysis of vRNA levels and clinical chemistry revealed noteworthy findings in animals vaccinated with NP+GP38, NP, and GP38. While no statistical differences were seen in vRNA levels in tissues or mucosal samples across groups, we detected higher copy numbers of CCHFV RNA in the blood of GP85- and GP160-vaccinated animals than in GP38-vaccinated animals (Fig. 5A). Next, alterations in clinical chemistry parameters were investigated. In NP- and NP+GP38-vaccinated animals, blood calcium, chloride, and sodium levels were significantly less altered than in mock-vaccinated animals (Fig. 5B). While alkaline phosphatase (ALP) levels were lower in NP+GP38- and GP38-vaccinated animals, ALP, alanine aminotransferase (ALT), and aspartate aminotransferase (AST) levels were less altered in GP38-vaccinated group than in GP85- and GP160-vaccinated groups (Fig. 5B and Supplementary Fig. 2 for the rest of the panel). Humoral immune responses remained comparable to those seen on day of challenge (D0) in all vaccine groups (Supplementary Fig. 3).

**Fig. 6:**
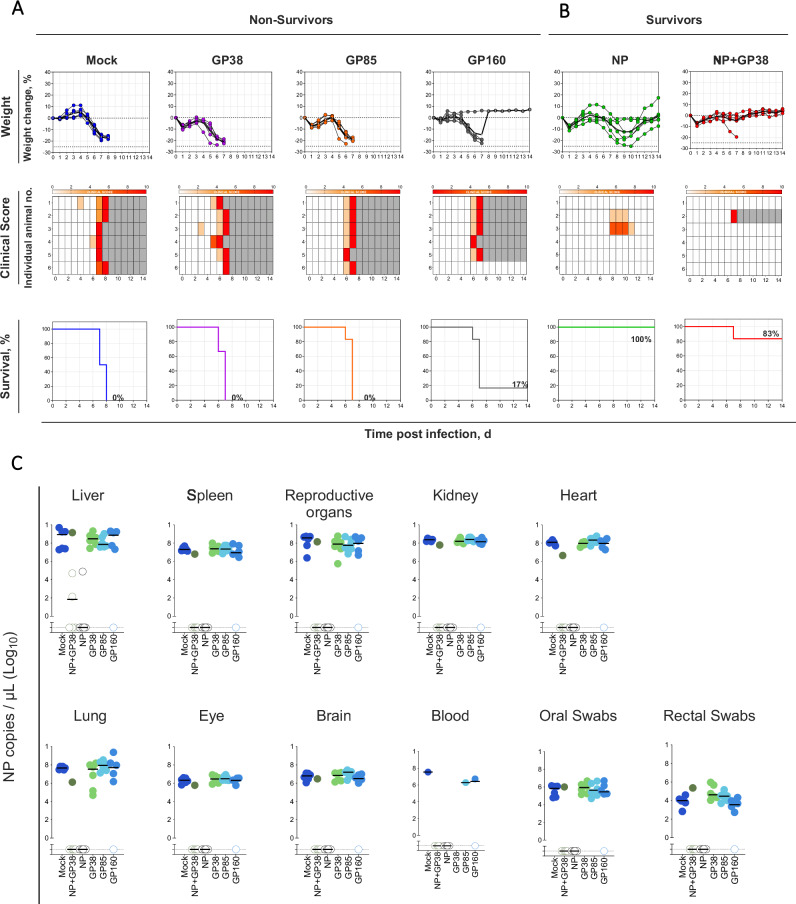
Vaccination with NP and NP + GP38 protects mice from challenge with a lethal dose of CCHFV. Two weeks after the second vaccination, animals were challenged with a lethal dose of CCHFV IbAr10200 and monitored for 14 days and weight changes (%), clinical scores, and survival (%) for **A** non-survivors (groups with <20% survival) and **B** survivors (groups with >20% survival) are given. Weight change is represented as percent decrease from baseline (taken on the day of challenge) for individual animals. Clinical scores (from 0–10) are depicted as increasing intensity of red. Gray boxes indicate end of monitoring. **C** Samples of various tissues collected when animals reached endpoint criteria from non-survivors (fatal; 6–8 dpc) and at study completion (14 dpc) from survivors. Blood samples could not be collected from the animals found dead. Viral shedding was determined by quantifying viral RNA from oral and rectal swabs collected when euthanasia criteria were met (non-survivors) and at study completion (survivors). Horizontal line represents the median value for the group. Multiple comparisons were performed using a two-way ANOVA. *p* values were adjusted for multiple comparisons using the two-stage linear set-up procedure of Benjamini, Krieger, and Yekutieli (*p < 0.05; **p < 0.001; ***p < 0.0003).

Following challenge, subgroups of mice were monitored for 14 days. All mock-vaccinated animals started to lose weight and showed clinical signs after 4 days post challenge (dpc), succumbing to infection 6–8 dpc. GP38-, GP85-, and GP160-vaccinated animals developed clinical signs similarly to mock-vaccinated animals and succumbed to infection, with only one survivor in the GP160 vaccine group (Fig. 6A). On the other hand, animals that were vaccinated with NP (6 out of 6) or NP+GP38 (5 out of 6) were protected from lethal outcome (Fig. 6B). All animals that succumbed to infection exhibited high viral loads in all examined tissues at the time of death (Fig. 6C). In survivors, viral RNA was detectable solely in liver samples from 2 animals in the NP+GP38 group and 1 in the NP vaccine group. Viral RNA was detectable in mucosal swabs collected at time of death (6–8 dpc) from mock-vaccinated and GP38-, GP85-, or GP160-vaccinated animals. Mucosal swabs collected from vaccinated survivors (NP, NP+GP38, and GP160 groups; 14 dpc) had no detectable vRNA. These findings underscore the protective efficacy of NP and NP+GP38 vaccine candidates against CCHFV.

Next, we investigated the effect of heterologous challenge on immune responses. ELISA was performed with GP38 from CCHFV Turkey, Hoti, and IbAr10200 strains and plasma samples from mice previously infected with CCHFV Turkey or IbAr10200 or vaccinated with Hoti GP38. Antibodies induced by Hoti GP38 vaccination displayed similar endpoint titers when probed with Hoti and Turkey GP38 proteins (Supplementary Fig. 4A, B). However, lower endpoint titers were detected when probed with IbAr10200 GP38 protein (Supplementary Fig. 4C), indicating that Hoti GP38-induced antibodies bind the challenge strain more poorly.

### Morbidity reduction by the addition of GP38 antigen to NP vaccine

Both NP- and NP+GP38-vaccinated animals were protected from lethal outcome, with 6/6 (100%) survivors in NP group and 5/6 (83%) survivors in NP+GP38 group. GP38 also alleviated clinical signs in survivors. In NP-vaccinated animals, based on weight loss from baseline at D0, disease signs were delayed compared to mock-vaccinated animals (Supplementary Table 1); only 2 animals had clinical scores ≥2, indicating the absence of clinical signs for most of the NP-vaccinated animals (Fig. 6B). All animals recovered from disease and began to regain weight ~10 dpc. In contrast, in the NP+GP38 vaccination group, the survivors lost less weight (2.3%) than NP-vaccinated (15.9%) or mock-vaccinated (17.5%) animals (calculated from maximum weight loss of individual animals from D0 baseline; Fig. 6A, B).

**Fig. 7:**
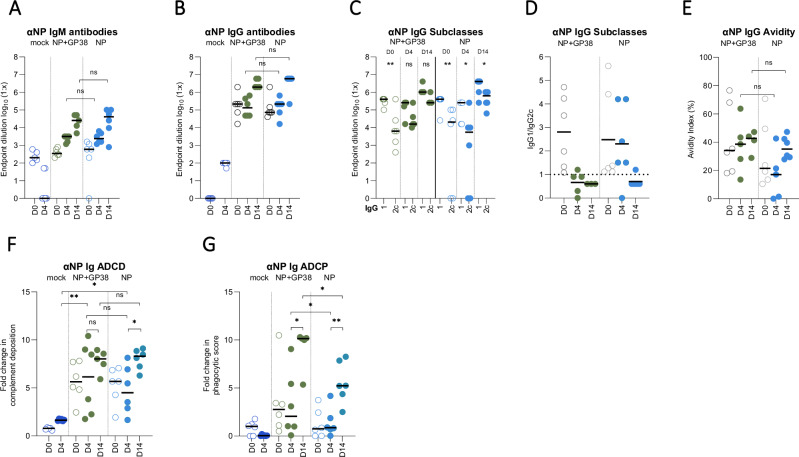
Anti-NP humoral immune responses to CCHFV challenge. NP-specific IgM **A** and IgG **B** responses 4 and 14 days post challenge (dpc) were determined as endpoint titers in plasma samples. **C** IgG1 and IgG2c titers were determined as endpoint titers in plasma samples. **D** IgG1 to IgG2c ratios were determined by dividing the endpoint titers of IgG1 by endpoint titers of IgG2 from individual animals. The dotted horizontal line represents IgG1:IgG2c ratio = 1. **E** Avidity indices of anti-NP antibodies of vaccinated animals were determined 4 and 14 dpc. For the avidity of IgG antibodies, AUC of 7-point dilutions of urea-treated and untreated samples were determined, and the avidity index was calculated as follows: (AUC of the urea-treated sample/AUC of untreated sample) × 100. Each dot on the graphs represents the mean value of the replicates from each animal and the horizontal line represents the median value for the group. ADCD function of anti-NP **F** antibodies represented as the fold change in complement deposition, and ADCP function **G** represented as fold change in phagocytic score over mock-vaccinated animals of corresponding timepoint 0 and 4 dpc. Results at 14 dpc were represented as fold change over 4 dpc mock-vaccinated animals. All samples were tested in duplicate. Two-tailed nonparametric *t* test and ordinary one-way ANOVA (*p < 0.05; **p < 0.001; ***p < 0.0003) were used for statistical analyses when applicable. Statistical analyses for IgM and IgG titers were conducted between plasma samples collected 4 and 14 dpc from NP + GP38-vaccinated and NP-vaccinated animal samples to compare endpoint titers. For IgG subclasses, statistical analyses were performed using IgG1 and IgG2 titers of individual vaccine groups in plasma samples collected on 0, 4, and 14 dpc. For IgG avidity, statistical analyses were performed between plasma samples of NP + GP38-vaccinated and NP-vaccinated animals collected 4 and 14 dpc.

At the study endpoint (14 dpc), all surviving animals in NP+GP38 and NP groups exhibited comparable anti-NP IgM and IgG antibody titers (Fig. 7A, B). These antibodies demonstrated similar IgG1 to IgG2c ratios and avidity (Fig. 7C–E). On day 14, both groups had anti-NP antibodies with similar ADCD function, and the NP+GP38 group had significantly higher ADCP function (Fig. 7F, G).

**Fig. 8:**
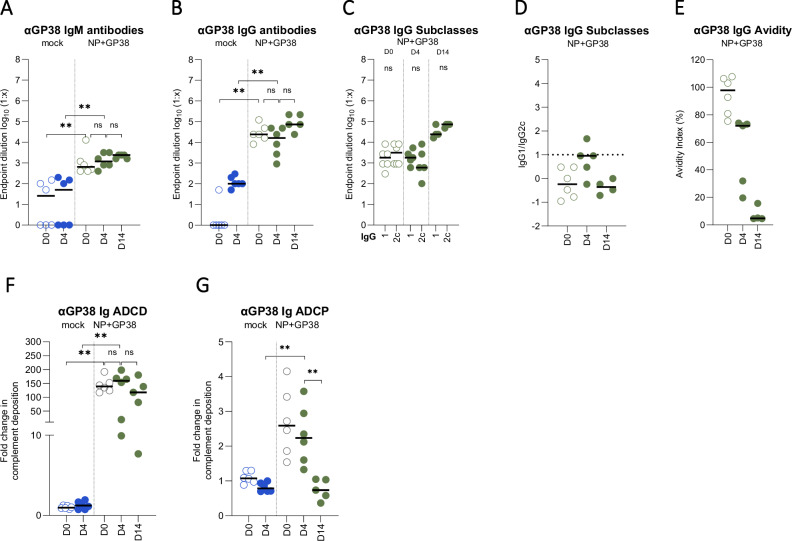
Anti-GP38 humoral immune responses to CCHFV challenge. GP38-specific IgM **A** and IgG **B** responses 4 and 14 dpc were determined as endpoint titers in plasma samples. **C** IgG1 and IgG2c titers were determined as endpoint titers in plasma samples. **D** IgG1 to IgG2c ratios were determined by dividing the endpoint titers of IgG1 by IgG2 titers in samples from individual animals. Dotted horizontal line represents IgG1:IgG2c = 1. **E** The avidity indices of anti-GP38 antibodies of vaccinated animals 4 and 14 dpc were determined. For the avidity of IgG antibodies, AUC of 7-point dilutions of urea-treated and untreated samples were determined, and the avidity index calculated as follows: (AUC of the urea-treated sample/AUC of untreated sample) × 100. Each dot on the graphs represents the mean value of the replicates from each animal and the horizontal line represents the median value for the group. ADCD function of **F** anti-GP38 antibodies represented as the fold change in complement deposition, and ADCP function **G** represented as fold change in phagocytic score over mock-vaccinated animals of corresponding timepoint 0 and 4 dpc. Results at 14 dpc were represented as fold change over 4 dpc mock-vaccinated animals. All samples were tested in duplicate. Two-tailed nonparametric *t* test and ordinary one-way ANOVA (*p < 0.05; **p < 0.001; ***p < 0.0003) were used for statistical analyses when applicable. Statistical analyses for IgM and IgG titers were conducted between plasma samples collected 0, 4 and 14 dpc from NP + GP38-vaccinated animal samples to compare endpoint titers. For IgG subclasses, statistical analyses were performed with IgG1 and IgG2 titers of individual vaccine groups in plasma samples collected 4 and 14 dpc. For IgG avidity, statistical analyses were performed between plasma samples collected 4 and 14 dpc from NP + GP38-vaccinated animals.

NP+GP38-vaccinated animals had similar levels of anti-GP38 IgM and IgG on day 14 as on day 4 (Fig. 8A, B). Identifying IgG subclasses indicated more IgG2c post infection (Fig. 8C, D), indicative of a shift toward Th1-biased responses when animals are progressing toward convalescence. ADCD activity of these antibodies was similar on days 14 and 4 (Fig. 8F), but avidity and ADCP activity were lower by day 14 (Fig. 8E, G).

### Pathology and cytokine responses in vaccine-protected groups

Since minimal weight loss was observed after NP+GP38 vaccination compared to NP vaccination, we analyzed the pathology of the liver and spleen after 4 and 14 dpc for these groups (Supplemental Table 2). Generally, liver and spleen pathology were similar in NP+GP38-, NP-, and mock-vaccinated animals 4 dpc. Most livers showed no changes or scattered small foci of hepatocyte necrosis and inflammation associated with rare viral antigen detection by immunohistochemistry (IHC) (Supplementary Fig. 5A); livers from 2 NP-vaccinated animals also had discrete foci of ischemic necrosis. Spleens from mock-vaccinated animals showed minimally increased numbers of macrophages and neutrophils in the red pulp and rare IHC staining, while most spleens of NP+GP38- or NP-vaccinated animals had only lymphoid reactivity without IHC staining (Supplementary Fig. 5A). Liver and spleen from two out of six in the NP+GP38 group showed more pathology and more IHC staining, with severe changes in one animal (Supplementary Fig. 5B). Correlating with this observation, viral RNA was also detectable in these two animals (Fig. 5A).

**Fig. 9:**
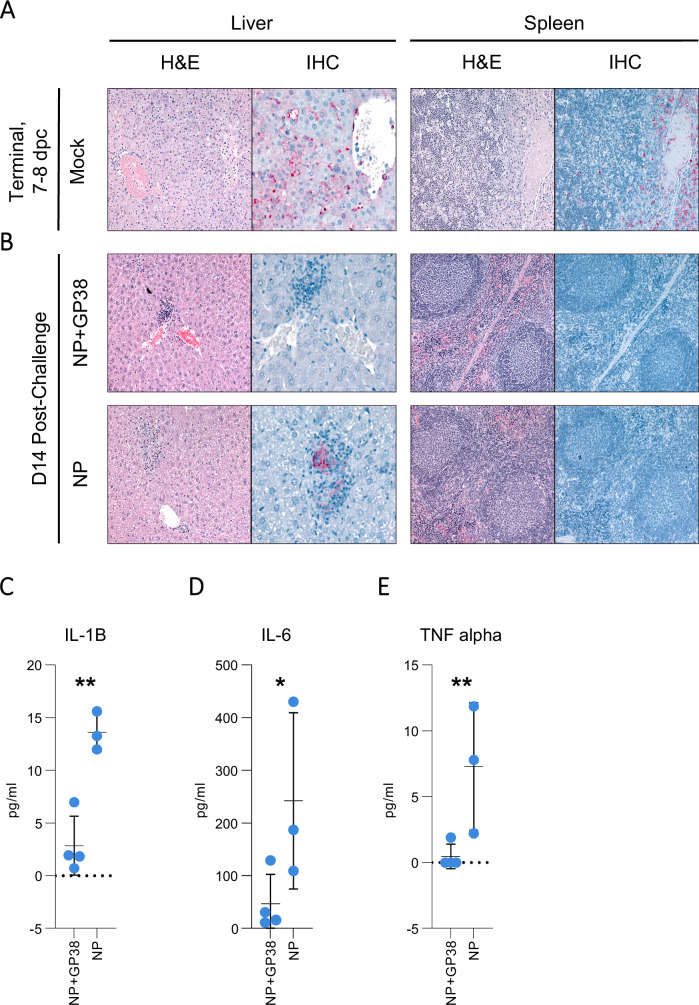
Liver and spleen pathology, CCHFV antigen detection, and pro-inflammatory cytokine levels in vaccine-protected animals. Tissue specimens were stained with hematoxylin and eosin (H&E), and antigen detection was visualized by IHC using rabbit polyclonal serum directed against CCHFV nucleoprotein. Representative images of histopathological findings in liver and spleen, and CCHF antigen detection by IHC are shown. **A** Mock-vaccinated animals when euthanasia criteria were met had extensive hepatocyte necrosis and CCHF antigen detection by IHC (red) in hepatocytes, intravascular leukocytes, and endothelial cells. Spleens had lymphoid reactivity and necrosis/apoptosis, with macrophage infiltration and extensive IHC staining (red). **B** Survivors in NP and NP + GP38 vaccine groups collected 14 dpc had scattered foci of inflammation with minimal or no hepatocyte necrosis and no or rare IHC staining (red). Spleens showed lymphoid reactivity without IHC staining. **C** IL1-B, **D** IL-6, and **E** TNF-alpha levels from plasma samples of surviving animals were determined by using ProcartaPlex Mouse Th1/Th2 Chemokine panel. Results are represented in pg/mL. Horizontal line represents the median value for the group. Statistical analyses were performed using non-parametric one-tailed Mann–Whitney *U* tests to compare cytokine levels (*p = 0.0571; **p = 0.0286).

In contrast to the mild pathology observed in all groups 4 dpc, livers of mock-vaccinated animals at the time of death or euthanasia (7–8 dpc) displayed widespread hepatocyte necrosis and acute inflammation with extensive IHC staining (Fig. 9A). Spleens showed variable prominent lymphoid apoptosis/necrosis, with neutrophil and/or macrophage infiltration and extensive IHC staining. However, most livers from vaccinated survivors collected 14 dpc showed minimal necrosis similar to that seen on day 4, with rare or no IHC staining. Liver inflammation in vaccinated survivors comprised lobular mononuclear infiltrates, and 3 of 5 livers of NP+GP38-vaccinated survivors had only minimal pathology; none of the 5 showed IHC staining, while 5 of 6 livers from NP-vaccinated animals had more than just minimal changes and positive IHC staining (Supplementary Table 2). Vaccinated survivors showed splenic reactivity lower than that from 4 dpc and without lymphoid depletion or macrophage infiltration and without IHC staining (Fig. 9B).

Next, we investigated plasma cytokine levels in survivors at 14 dpc. Only IL1-B, IL-6, and TNF-alpha were detected at higher levels in NP-vaccinated animals than in NP+GP38-vaccinated animals (Fig. 9C–E, and Supplementary Fig. 6 for comparative cytokine levels on days 0, 4 and 14).

We investigated antibody titers to Gc, a viral antigen not present in any of the vaccines, to rule out any contribution of the humoral immune response to this protein to the outcome, and no significant differences were observed between groups on D4 (Supplementary Fig. 7A, B) and D14 (Supplementary Fig. 7C, D).

Collectively, these data support that morbidity in vaccine-protected animals is associated with an increase in liver pathology and proinflammatory cytokines in NP-vaccine-protected animals and that the challenge resulted in no measurable differences in antibody responses to Gc, an antigen absent in all vaccines tested.

## Discussion

**Fig. 10:**
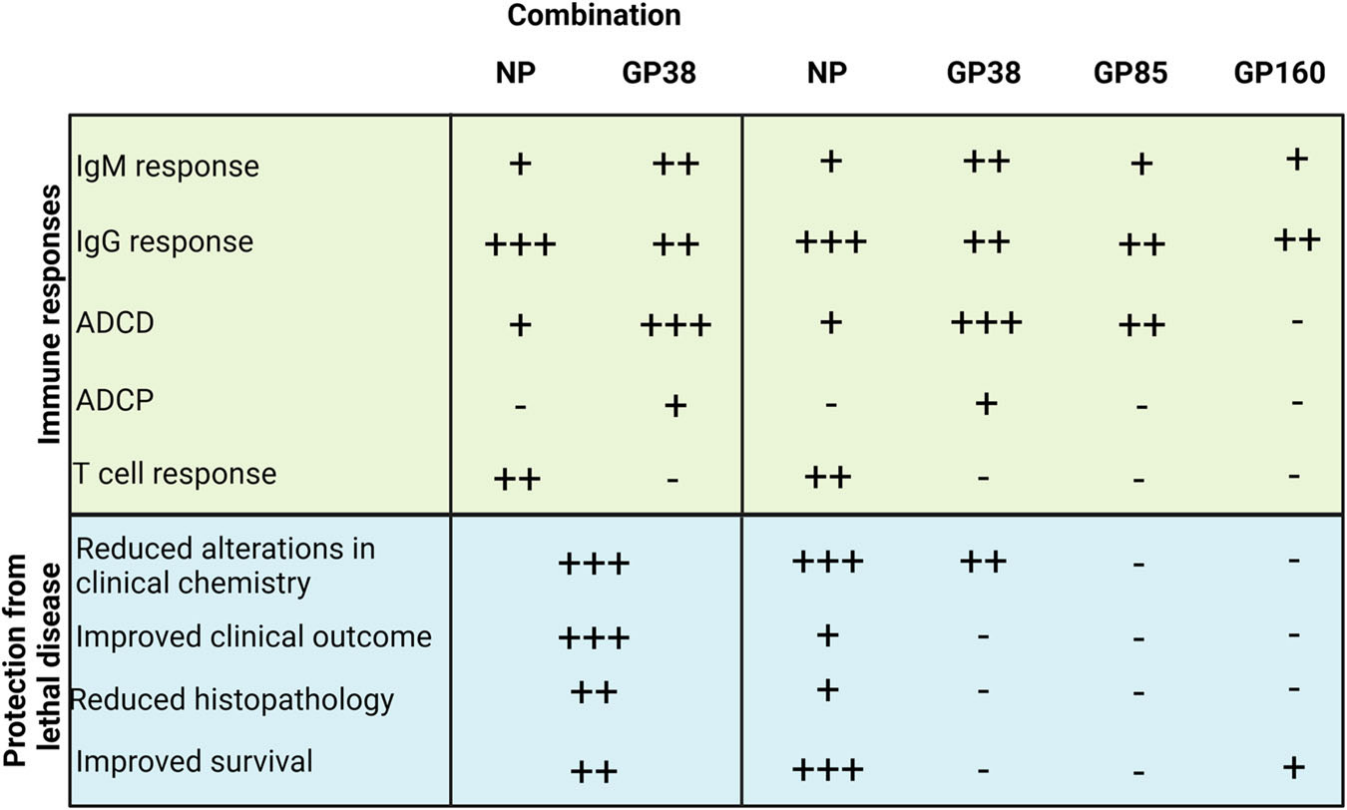
Qualitative representation of outcomes of vaccination with recombinant CCHFV proteins. Schematic summary of the immune responses and protective efficacy of each vaccine. IgM and IgG responses, ADCD, and ADCP functions of antibodies and T-cell response were used as indicators of immune response. Clinical chemistry profiles, improved clinical outcome, reduced histopathology of liver and spleen, and survival were used as indicators of protective efficacy. To describe the strength of humoral immune responses and protection from lethal disease, the following scale was used: absent (−), mild (+), moderate (++), and strong (+++). Graphical illustrations were prepared with BioRender (www.biorender.com).

Developing vaccines that are efficacious, cost-effective, and rapid to produce is an urgent need for mitigating the current impact and future risks posed on public health by orthonairoviruses. In this study, we demonstrate that immunizing mice with recombinant NP induces strong humoral immune responses with dominant IgG1 subclass, moderate avidity, and ADCD function. NP vaccination also induced cellular immune responses and resulted in 100% survival following heterologous challenge in a lethal mouse model. Immunization with GP38, GP85, or GP160 alone was insufficient to confer significant protection. Importantly, adding GP38 to NP vaccination resulted in a better outcome than NP alone by reducing morbidity in survivors (Figs. 6B and 10).

Many vaccine studies have been conducted to understand immune correlates of protection from CCHFV infection and to develop effective countermeasures. Knowledge gained from some of these studies shows that neutralizing antibodies are not necessarily associated with or required for protection from CCHF in small animal models^26,27^. While neutralizing antibodies work by preventing the virus from infecting host cells, non-neutralizing antibodies offer other mechanisms that can also ameliorate disease outcome. Vaccines inducing non-neutralizing antibodies have been shown as protective against other diseases; effector functions like antibody-dependent cellular toxicity, phagocytosis, NK activation, and complement deposition were associated with protection in these models^58–62^. Vaccines and therapeutic non-neutralizing monoclonal antibodies targeting CCHFV NP and GP38 have also been shown to confer protection, indicating that eliciting non-neutralizing antibody responses is a promising approach for developing vaccines against CCHFV^40,44,45,47,63^.

The efficacy of vaccine platforms utilizing CCHFV NP in inducing humoral and cellular responses has been studied, with some evaluated in challenge studies. Despite inducing NP antibodies, a modified vaccinia Ankara-vectored vaccine did not confer protection^64^. On the other hand, DNA, RNA, and some other viral vector vaccines conferred protection against homologous and heterologous challenge^40–43,65,66^. Here, we observed a robust antibody response to recombinant NP, characterized by a dominant IgG1 subclass and ADCD. The dominant IgG1 subclass was also observed in vaccine studies using bovine herpesvirus type 4 and adenovirus type 5 vectors, as well as an mRNA vaccine expressing CCHFV Ankara-2 strain NP^43,66^. While these vaccinations led to varying levels of protection from lethal outcome, surviving animals lost 10–20% of their starting body weight, similarly to our NP-vaccinated group. In contrast, vaccine candidates with actively replicating RNA genomes, such as an alphavirus-based replicon RNA vaccine expressing CCHFV NP (repNP)^40^ and CCHF viral replicon particle (VRP)^63^ expressing CCHFV L-RNA polymerase and NP conferred protection without weight loss or clinical signs of disease. Although comparing the outcomes of these vaccines is challenging due to the differences in study designs, the improved profile of VRP and repNP vaccines over other platforms suggests a contribution of Fc-mediated functions of the anti-NP antibodies in mitigating infection and clinical signs. Both replicating RNA vaccine platforms resulted in dominant IgG2c and IgG2b responses over IgG1, indicative of Th1-biased immune responses. Additionally, the VRP vaccine induced anti-NP antibodies with superior Fc-mediated functions compared to this study, which suggest that the dominant IgG2c profile is associated with enhanced Fc-mediated functions and improved control of the disease. Future research should address the potential association between vaccine efficacy and the development of an NP-induced Th1 response.

Another distinction was observed in cellular immune responses to these vaccinations. T cell responses, while significant, were ~3-fold weaker when using subunit vaccines than repNP and VRP vaccines^40,63^. While the contribution of humoral and cellular immune responses to recovery from CCHFV infection is still unknown for the NP subunit vaccine, this study further emphasizes the value of CCHFV NP as a promising vaccine antigen and relevance of delivering NP antigen as a recombinant protein. The high sequence conservation of CCHFV NP among diverse strains is advantageous, as an NP vaccine is more likely to retain its efficacy against most strains and to have broader geographical utility. Few CCHFV candidate vaccines have been evaluated in heterologous challenge models. In the repNP vaccine model, CCHFV strain Hoti (Europe I) NP sequence was used for the vaccine constructs, while the challenge strain was CCHFV UG3010 (Africa II); this vaccine conferred heterologous protection^40^. In the CCHF VRP vaccine, the S segment (NP encoding segment) was derived from CCHFV IbAr10200 (Africa III), and the vaccine conferred protection when animals were challenged with CCHFV Turkey (Europe I) and Oman 97 (Asia I)^67^. In addition, NP mAb 9D5 protected mice from homologous IbAr10200 (Africa III) and heterologous Afghan09-2990 (Asia I) challenges^56^. In our study, recombinant NP was derived from the CCHFV Hoti strain (Europe I), and the challenge strain was from Africa III clade^68^. This body of work emphasizes that the CCHFV NP antigen is a prime candidate for the future development of a broadly protective CCHFV vaccine.

CCHFV GP38 is another protective antigen derived from the proteolytic processing of the GPC. DNA vaccines encoding the complete GPC or GP38 or anchoring GP38 in inactivated rabies virus particles represent other effective vaccine strategies^38,44^. Certain anti-GP38 monoclonal antibodies targeting site 1 are protective in mice, demonstrating the role of particular anti-GP38 antibodies in protection^45–47^. When we evaluated GP38 as a subunit vaccine, the humoral immune responses resulted in a balanced IgG1/IgG2c response, with antibodies showing Fc-mediated functions. The vaccination did not result in any detectable GP38-specific T-cell responses. Similarly to the subunit vaccine, no T cell response was detected when using the rabies-particle vaccine displaying GP38, but there was a notable difference in humoral responses^44^. Even though both studies utilized the same adjuvants, humoral immune responses were more Th1-biased when using the rabies-particle vaccine than when using the subunit vaccine. Unlike the GP38 subunit vaccine, the rabies-particle vaccine conferred complete protection from lethal challenge^38,44^.

Another important distinction between these studies was the challenge strategy. In DNA and rabies-particle vaccine studies, CCHFV IbAr10200 was selected both for vaccine constructs and the challenge strain^38,44^, while in the subunit vaccine study, the vaccine antigen was from the CCHFV Hoti strain (Europe I clade) and the challenge strain was CCHFV IbAr10200 (Africa III clade). We observed lower cross-reactivity between GP38 subunit vaccine-induced anti-Hoti GP38 antibodies and IbAr10200 GP38 antigen; this reduced affinity may have contributed to the lack of protective efficacy of GP38 as a standalone subunit vaccine. Future studies should address if GP38 antibodies generated by other vaccine platforms are sufficient to confer broad protection against genetically divergent strain of CCHFV.

Vaccination with GP38, GP85, or GP160 individually did not confer significant protection. Notably, GP38 vaccination resulted in fewer alterations in clinical chemistry than mock-, GP85- and GP160-vaccination, and less vRNA was detected in blood than in GP85- and GP160-vaccinated animals 4 dpc. These discrepancies in disease outcomes might have resulted from differences in humoral immune responses. Overall, when GP38 without MLD was presented to the immune system, IgG antibody subclasses were balanced, and these antibodies had Fc-mediated functions and better avidity. On the other hand, using MLD-containing GP38 (GP85 and GP160) resulted in dominant IgG1 subclasses with little or no Fc-mediated function and lower avidity. Like CCHFV GP160 and GP85, Ebola virus GP also contains a heavily *N-* and *O-*glycosylated, highly variable MLD^69^. The Ebola virus GP MLD shields GP epitopes and is cleaved by cathepsins upon entry to allow receptor binding to the intracellular receptor NPC1^70^. Thus, these MLD domains are proposed to protect functional epitopes from antibody action^70^. A similar shielding effect might explain the disparities between vaccine groups in our study.

Although vaccination with GP38 alone did not protect animals from lethal infection, combining GP38 with NP resulted in better clinical outcomes in surviving animals. Addition of GP38 prevented weight loss and reduced levels of proinflammatory cytokines IL-1β, IL-6, and TNF-alpha. Elevated concentrations of proinflammatory cytokines can contribute to tissue injury, and certain proinflammatory cytokines, particularly TNF-alpha and IL-6, have been associated with severe disease in CCHFV-infected humans^71–73^, while TNF-alpha signaling is associated with CCHFV pathogenesis in mice^74^. Accordingly, liver injury and antigen levels were limited 14 days after NP+GP38 vaccination, further emphasizing the benefits of adding GP38 to NP vaccination. In the cynomolgus macaque model, using both CCHFV GPC and NP as vaccines also better controlled viral replication, reduced signs of clinical disease, and reduced viral burden in liver^75^. While further studies are needed to confirm these observations, the positive effect of adding GP38 to NP vaccination suggests that anti-GP38 immune responses led to better protection from CCHFV-induced liver injury in the NP+GP38 vaccine group.

Future studies should also explore new ways to improve vaccination methods to maximize the efficacy of the subunit vaccine or explore NP and GP38 combinations using other platforms to further improve their efficacy. Another possible improvement to CCHFV vaccines is combining chemokines with vaccine antigens to improve dendritic cell antigen presentation, resulting in enhanced immune responses and germinal center reactions^76^. Nanoparticle formulations also successfully increase antigen deposition in lymph node resident follicular dendritic cells and germinal centers and enhance T cell immunity^77–80^. Using adjuvants that affect the strength and breadth of immune responses, such as increasing antibody avidity, memory B cell proliferation, and CD4^+^ T cell responses, may further improve the efficacy of the subunit vaccine^81,82^.

In summary, we demonstrate that a recombinant NP subunit vaccine represents an effective strategy for reducing CCHFV-induced mortality. Significantly, combining NP with GP38 yields better clinical outcomes in surviving animals. The high sequence conservation and protective efficacy of NP, along with GP38's clinical disease-ameliorating effect, position this vaccine strategy as a valuable solution for mitigating CCHF disease. This study establishes a crucial foundation for advancing CCHFV subunit vaccine platforms and for improving the antigen composition of other vaccine candidates utilizing alternative platforms and delivery methods.

## Methods

### Production of vaccine antigens

All the antigens used for immunization are derived from CCHFV Hoti sequences (GenBank: AW63616.1 and AWX63617.1). The sequence for expressing CCHFV nucleoprotein with N-terminal His and GST tags and HRV 3C site was optimized for bacterial expression and cloned into pET28a by Twist Bioscience (Supplementary Fig. 1A). The construct was transformed in *Escherichia coli* BL21 (DE3) strain (Thermo Fisher), and bacterial culture was grown in Luria broth with kanamycin. The culture was induced with 1 mM isopropyl β-D-1-thiogalactopyranoside when the optical density was 0.4–0.6. Following induction, the culture was transferred to 16 °C for overnight incubation. Cells were harvested by centrifugation, resuspended in lysis buffer (500 mM NaCl, 20 mM Tris-Cl [pH 7], 0.1% Triton-X, 5% glycerol, 1 mM MgCl_2_, 25 U/mL benzonase), and sonicated^55,56^. CCHFV NP was purified by nickel affinity chromatography (HisTrap Excel column; Cytiva) followed by size exclusion chromatography (SEC; Superdex 200 increase 16/300; Cytiva). The fractions with the lowest nucleic acid content were pooled and cleaved with HRV 3C protease at 4 °C overnight (AcroBiosystems). The flow-through of the second nickel affinity chromatography containing NP without the tags was further purified by SEC to collect monomeric NP (Supplementary Fig. 1B).

CCHFV Hoti GP160, GP85, and GP38 are products of the same construct containing the CCHFV Hoti strain MLD-GP38 sequence cloned into pEEV plasmid with a C-terminal His tag and Twin-StrepTag (Twist Bioscience)^13,14^ (Supplementary Fig. 1C). Proteins were expressed in Expi293F cells (Thermo Fisher) grown in Expi293 expression medium via transient transfection using FectoPro transfection reagent (Polypus) as described previously^47^. Cell culture supernatants were filtered through a 0.2-micron polyethersulfone membrane and loaded onto HisTrap Excel columns (Cytiva) for immobilized metal affinity chromatography. Proteins were further purified by SEC (Superdex 200 increase 16/600 GL; Cytiva), and fractions containing G160, GP85, and GP38 were collected and pooled separately. Running buffer for SEC was phosphate-buffered saline (PBS) for all purifications. Protein expression was confirmed using sodium dodecyl-sulfate polyacrylamide gel electrophoresis (SDS PAGE) (Supplementary Fig. 1D). Following confirmation, proteins were quantified, aliquoted, and stored at −80 °C. The same proteins were used to measure homologous antibody responses to vaccination.

### Additional antigens production for ELISA

To detect differences in the binding of antibodies to GP38 proteins from different CCHFV strains, CCHFV IbAr10200 GP38 and Turkey GP38 proteins (GenBank: AWX63620.1, NP_950235, ASW22359.1) were expressed as previously described^47^. Additionally, CCHFV Hoti Gc was expressed and purified as previously outlined^63^.

### Vaccine preparation

Purified proteins in PBS were mixed with equal volumes of squalene-based oil-in-water adjuvant by pipetting (AddaVax, Invivogen) to give 10 µg antigen and 5 µg pure synthetic MPLA (Invivogen) per vaccine dose. Animals vaccinated with GP85 and GP160 received doses containing the same molar concentration of GP38 as the GP38 alone vaccine group. The mock-vaccinated group received the same volume and concentration of the adjuvants prepared in PBS. In the combination group, animals received 10 µg of each antigen. Prepared vaccines were used immediately after preparation.

### Challenge virus

Heterologous challenge with recombinant CCHFV IbAr10200 was used to ensure uniform lethality in mice. Recombinant CCHFV IbAr10200, shown to be equivalent to the original isolate (IbAr10200; GenBank KJ648914, KJ648915, and KJ648913), was used as the challenge virus^17,83^. Stock and inoculum titers were calculated via indirect immunofluorescent TCID_50_ (Reed & Muench method) assay using BSR-T7/5 cells^84^.

### In vivo experiments

Groups of C57BL/6J mice (*n* = 6; 3 females and 3 males; 6 weeks of age) purchased from Jackson Laboratory (strain 000664) were vaccinated SC with 100 µL of subunit vaccines described above 28 (D-28) and 14 (D-14) days before the day of challenge (D0). Mock-vaccinated groups received adjuvant only in PBS. Six mice from each vaccination group were euthanized on D-14 and D0 to investigate immune responses following first and second vaccination. On D0, animals were immunosuppressed under isoflurane anesthesia with 2.5 mg/mouse mAb-5A3 (Leinco Technologies Inc.) in dorsal recumbency by the intraperitoneal (IP) route and then subsequently placed in ventral recumbency and infected with recombinant CCHFV IbAr10200 SC between the scapulae (backtiter dose: 1000 TCID_50_). A group of mice was serially euthanized 4 dpc to investigate disease progression and immune responses. The other group was monitored for 14 days after challenge for survival outcomes.

Plasma samples from animals infected with different CCHFV strains collected for other studies were used in ELISA assays to determine the binding of antibodies to GP38 from different phylogenic clades. Briefly, groups of C57BL/6J mice were immunosuppressed as described above and then subsequently placed in ventral recumbency and infected with 100 TCID_50_ CCHFV IbAr10200 and CCHFV Turkey-200406546 SC between the scapulae.

Blood samples were collected from infected animals and plasma was separated from whole blood collected in lithium heparin tubes by centrifuging 3 min at 8000 rpm. Samples were inactivated using gamma irradiation (5 million rads from a ^60^Co source).

Mice were group-housed in a climate-controlled laboratory with a 12 h day/night cycle on corn cob bedding (Bed-o'-Cobs 1/4”, Anderson Lab Bedding) with cotton nestlets in an isolator-caging system (Tecniplast GM500 cages) with a HEPA-filtered inlet and exhaust air supply; they were provided commercially available mouse chow and water ad libitum. Bedding, feed, and water were sterilized for the challenge phase. Mice were evaluated daily for clinical signs of disease and assigned a score ranging 0–10 based on the following criteria: piloerection, hunched posture, hypoactivity, percent weight loss, abnormal respiration, dehydration, and neurological signs (ataxia, paresis). Euthanasia criteria were met when weight loss exceeded 25% from baseline (day of infection) and/or the clinical score reached 10. Mice were humanely euthanized via isoflurane exposure followed by cervical dislocation at the serial timepoints indicated or when meeting euthanasia criteria according to protocols approved by CDC’s Institutional Animal Care and Use Committee (IACUC).

### RNA extraction and qRT-PCR

Tissue (~1 mm in thickness in one direction) was homogenized in 1 mL of MagMax lysis buffer concentrate. Whole blood (50 µL) collected in lithium heparin was added to 500 µL of MagMax lysis buffer concentrate. Viral RNA was extracted using the MagMax-96 Total RNA isolation kit (Thermo Fisher) on a 96-deep well KingFisher Apex System (Thermo Fisher) into 75 µL elution buffer. Samples were treated with Baseline-ZERO DNase (LGC Biosearch) as part of the extraction protocol.

All RT-qPCR assays were prepared in 384-well plates using the OT-2 Liquid Handler (Opentrons). Viral RNA was quantified using a primer/probe set targeting the NP ORF of the S genomic sequence using the SuperScript III Platinum One-Step RT-qPCR kit (Thermo Fisher). Viral RNA levels from harvested tissues and blood were normalized using the validated reference genes, *Ppia* and *Gusb* (Davies et al., submitted), and quantified with an RNA standard curve of known concentration. Data are reported as S genome copy number/µL RNA^85^.

### Clinical chemistry

Whole blood samples, when available, were collected in lithium heparin and analyzed on Piccolo Xpress chemistry analyzers (Comprehensive Metabolic Panel, Abaxis) within 1 h of collection.

### ELISA

#### IgM and IgG ELISA

Immulon 2HB plates were coated with 100 μL of 500 ng/mL antigen prepared in PBS and incubated overnight at 4 °C. Wells were washed 4× with 300 μL PBS-T (0.1% Tween-20 in PBS) and blocked (5% w/V non-fat dry milk in PBST) for 1 h at room temperature (RT). Following blocking, the buffer was decanted, and 100 μL of mouse plasma prepared in blocking buffer with twofold serial dilutions (range 1:200 to 1:409600) was added to the wells in duplicate. After 1 h incubation at RT, wells were washed 4×, anti-mouse IgG HRP (1:3000, Invitrogen, #61-6520) or anti-mouse IgM HRP (1:1000, Invitrogen, #31456) was added to the wells (100 μL), and plates were incubated for 1 h at RT. Following incubation, wells were washed 4×, and 100 μL TMB Ultra ELISA substrate (Thermo Fisher) was added and incubated for 10 min at RT. The reaction was stopped by adding ELISA stop solution (Thermo Fisher), and optical density was read at 450 nm on a Synergy Neo2 instrument (BioTek) microplate reader. A cut-off value was determined for each plate based on the average absorbance value of negative control wells plus 3 standard deviations. The highest dilutions with a signal above the determined cut-off value were assigned as the endpoint titers.

#### IgG subclass ELISA

Assay was performed as described above, and IgG1-specific (Abcam, #ab97240), and IgG2c-specific (Abcam, #ab97255) anti-mouse antibodies conjugated to HRP were used in 1:2000 dilution. The results were represented as an IgG2c to IgG1 ratio based on the endpoint titer of each sample.

#### Avidity ELISA

Assay was performed as described above with an additional treatment step using a chaotropic agent. Briefly, serum samples from individual animals were prepared in 3-fold dilution series in 4 replicates. Plates were incubated for 1 h as described above and washed 4× with PBST. Then, 200 µL/well PBS was added to 2 of these replicates (untreated), while 200 µL/well 6 M urea was added to the other 2 replicates (treated). After 10 min incubation at RT, plates were washed 4× times with PBST, and the rest of the assay was completed as described for IgG ELISA. The area under the curves were calculated for both untreated and treated replicates, and the avidity index was determined by dividing the area under the curve of the urea-treated wells by that of the PBS-treated wells, and then multiplying by 100^86^.

### Antibody functionality assays

#### ADCD

The assay was adapted from^87^. Recombinant CCHFV Hoti strain NP and GP38, purified as described above, were biotinylated (21435, EZ-Link™ Sulfo-NHS-LC-Biotinylation Kit) and coupled to 1.0 µm fluorescent red neutravidin microspheres (Thermo Fisher F8775). Excess antigen was removed by washing twice with PBS containing 5% BSA and collecting beads by centrifuging (20 min at 3000 × *g*). Antigen-coated beads were incubated with 10-fold diluted mouse plasma (2 h at 37 °C) and then washed twice with PBS (15 min at 2000 × *g*). Guinea pig complement (Cedarlane, CL4051) 1:25 diluted in gelatin veronal buffer (CompTech B102) was added and incubated for 15 min at 37 °C. Immune complexes were washed twice with 15 mM EDTA in PBS, followed by centrifugation (15 min at 2000 × *g*) and incubated for 15 min at RT with FITC-conjugated goat IgG fraction to guinea pig complement C3 (MP Biomedicals, 0855385), diluted 1:100 in PBS. Immune complexes were washed twice with PBS, followed by centrifugation (15 min at 2000 × *g*), and were analyzed on a Guava Cytometer. The results are represented as fold change in complement deposition over mock-vaccinated mouse plasma.

#### ADCP

The assay was adapted from^88^. Immune complexes were formed as described for ADCD, except biotinylated antigen was coupled to 1.0 µm fluorescent green neutravidin microspheres (Thermo Fisher F8776). Excess antigen was washed away with PBS containing 5% BSA. Antigen-coated beads were incubated with 10-fold diluted mouse plasma (2 h at 37 °C) and then washed twice with PBS (15 min at 2000 × *g*). Immune complexes were incubated overnight at 37 °C with 1 × 10^4^ THP1 cells per well. Cells were washed with PBS (5 min at 500 × *g*) and analyzed on a Guava Cytometer the next day. The phagocytic score was calculated by multiplying the percentage of bead-positive cells by the overall median fluorescence intensity and dividing that by 10,000. The results are represented as fold change in phagocytic score over mock-vaccinated mouse plasma.

### IFN-γ ELISpot assay

Single-cell splenocyte suspensions were prepared from euthanized animals using a tissue dissociator (GentleMACS, Miltenyi Biotec). Red blood cells (RBC) were lysed with 3 mL of RBC lysis buffer/spleen (Roche, 63354600) by incubating 5 min at RT. Cells were washed, counted, and seeded 2 × 10^5^ cells/well of Mouse-IFN-gamma ELISpot plates (Mabtech). The CCHFV 10200 NP peptide library (AbClonal 15-mers, 11 AA overlap) was prepared in 4 pools; the CCHFV Hoti MLD-GP38 peptide library (GenScript 15-mers; 9 AA overlap) was also divided into 4 pools, and 10 µg/mL peptide was used as stimulant. PMA was used as a positive control, and DMSO as a negative control. Per manufacturer's instructions, splenocytes were incubated for 48 h before spot development. Spots from each animal were counted using a CTL ELISpot reader, and background reactivity was subtracted and normalized to PMA-induced positive control wells.

### Cytokine/chemokine analysis

Plasma samples from each animal were gamma-irradiated (5.0 × 10^6^ rad dose) and analyzed using the ProcartaPlex Mouse Th1/Th2 Cytokine and Chemokine 20-plex panel according to manufacturer’s instructions (Thermo Fisher, EPX200-26090-901). Briefly, magnetic bead mixes were added to the wells, and a 4-fold dilution of the standards was prepared and added to the wells in duplicates. Universal assay buffer was used as background control. 25 µL of plasma samples were mixed with 25 µL universal assay buffer and added to the wells. After overnight incubation, detection antibody mixture was added, and plates were run on the Luminex xMAP Intelliflex System. The results were analyzed using ProcartaPlex Analysis App (Thermo Fisher Scientific). Five-parameter logistic algorithm was selected for best curve fit for the standards, and the concentrations of the samples were calculated by plotting expected concentrations of the standards against mean fluorescence intensity generated by each sample.

### Histology and immunohistochemistry

Liver and spleen specimens were fixed in 10% neutral buffered formalin and gamma-irradiated (2 × 10^6^ rad). Tissues were routinely processed for paraffin embedding, sectioning, and staining with hematoxylin and eosin. For the IHC assays, slides were stained with rabbit anti-CCHFV NP pAb (IBT Bioservices, #04-0011) diluted 1:1000, as previously described^89^. Livers were scored semi-quantitatively for hepatocellular necrosis and lobular inflammation, where 0 = not present, 1 = minimal, 2 = mild, 3 = moderate, 4 = severe. Spleens were scored semi-quantitatively for lymphoid changes, where 0 = not present, 1= mild reactive changes, 2 = moderate reactive changes, 3 = prominent reactive changes, 4 = lymphoid necrosis/apoptosis. Spleens were also scored for red pulp infiltration by neutrophils and macrophages, where 0 = not present, 1 = minimal, 2 = mild, 3 = moderate, 4 = severe. CCHF immunostaining was scored in liver and spleen, where 0 = no staining, 1 = rare, 2 = multifocal/mild, 3 = multifocal/moderate, 4 = extensive.

### Biosafety and ethics statement

All CCHFV infections were performed in biosafety level 4 facilities at the Centers for Disease Control and Prevention (CDC; Atlanta, GA, USA) following established standard operating procedures approved by the Institutional Biosafety Committee. All recombinant virus work was approved by the CDC Institutional Biosafety Committee. All animal procedures were approved by the CDC Institutional Animal Care and Use Committee (IACUC protocol 3342SPEMOUC) and conducted in accordance with the Guide for the Care and Use of Laboratory Animals. The CDC is fully accredited by the AAALAC-International.

### Statistical analyses

All statistical analyses were conducted using GraphPad Prism version 10.0.0. Two-tailed nonparametric *t* test and ordinary one-way ANOVA (*p < 0.05; **p < 0.001; ***p < 0.0003) were used for statistical analyses of ELISA, Fc-mediated function assays, and clinical signs. For PCR results, multiple comparisons were performed using a two-way ANOVA. *p* values were adjusted for multiple comparisons using the two-stage linear set-up procedure of Benjamini, Krieger, and Yekutieli. Non-parametric one-tailed Mann–Whitney *U* test was used to compare cytokine levels. The area under the curve in avidity ELISA was calculated using GraphPad Prism version 10.0.0.

### Supplementary information

Supplementary Material

## Data Availability

All relevant data are within the manuscript and its Supporting Information files.
