# Supplementary material for: Crimean Congo hemorrhagic fever virus nucleoprotein and GP38 subunit vaccine combination prevents morbidity in mice
Source: NPJ Vaccines. 2024 Aug 14;9:148. doi: 10.1038/s41541-024-00931-y (PMC11324950; doi:10.1038/s41541-024-00931-y)
Supplement: Supplementary file 1 — Supplementary Material [file 41541_2024_931_MOESM1_ESM.pdf]

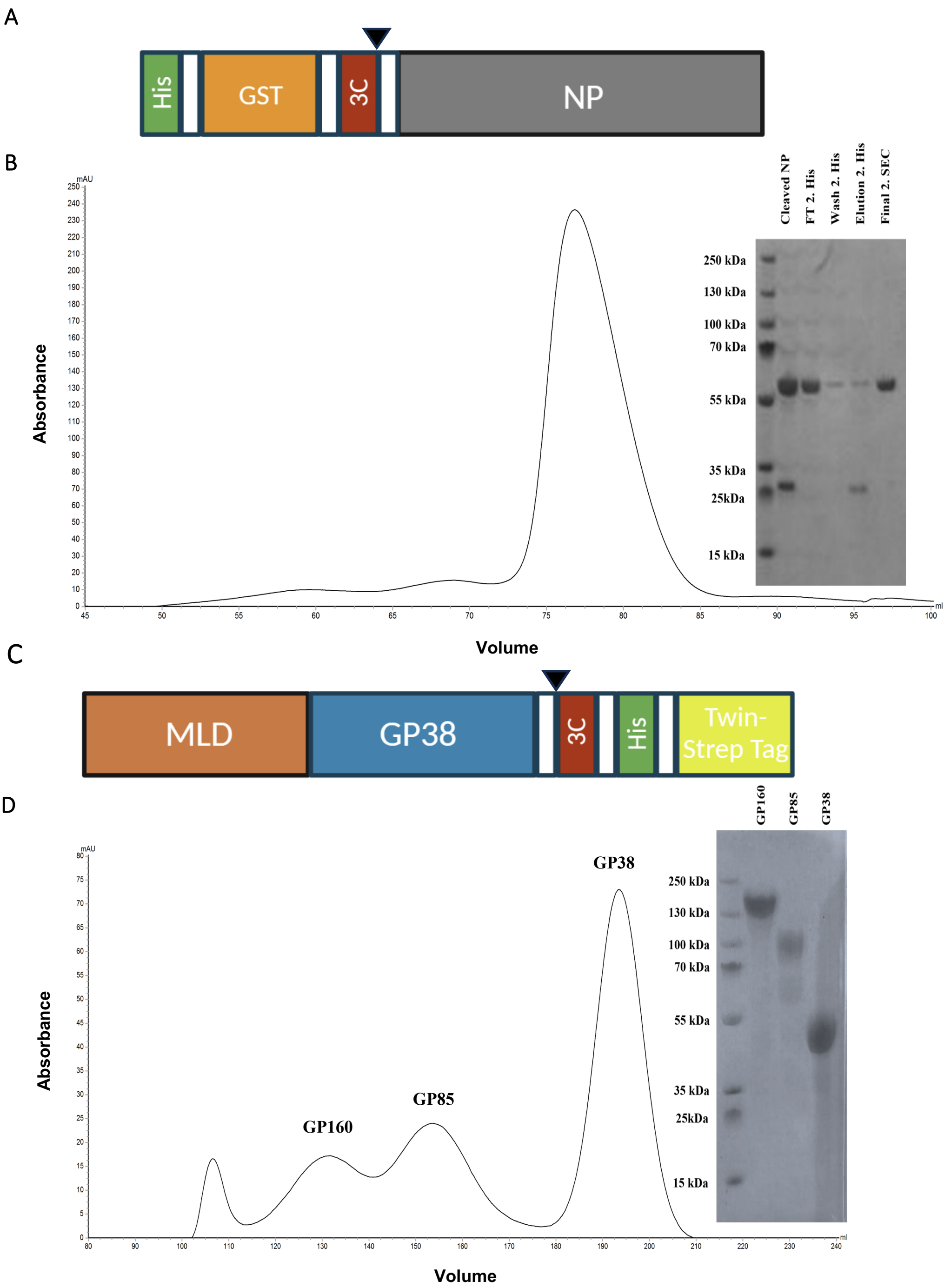

Supplementary Figure 1: Expression and purification of recombinant proteins used as vaccine antigens. (A) Schematic representation of the construct for expressing CCHFV Hoti NP. (B) Size exclusion chromatogram of the run performed to purify cleaved NP. SDS-Page shows the purification steps. Lane 1: ladder (Thermo Scientific, Pageruler prestained protein ladder, 26617; lane 2: cleaved NP before second His purification; lanes 3, 4, and 5: flow-through and elution of the second His purification; lane 6: sample from the pooled fractions from the second size exclusion chromatography. (C) Schematic representation of the construct for expressing CCHFV Hoti GP38 and GP38-containing proteins. (D) Size exclusion chromatogram of the run performed to separate GP160, GP85, and GP38. SDS-PAGE shows migration of samples from each peak. Lane 1: ladder; lane 2: GP160; lane 3: GP85; lane 4: GP38. Arrowheads show the cleavage sites to remove tags, and GS linkers are indicated in white. X-axis indicates volume, and Y-axis represents absorbance in milli-absorbance units (mAU) in chromatograms. Graphical illustrations were prepared with BioRender (www.biorender.com).

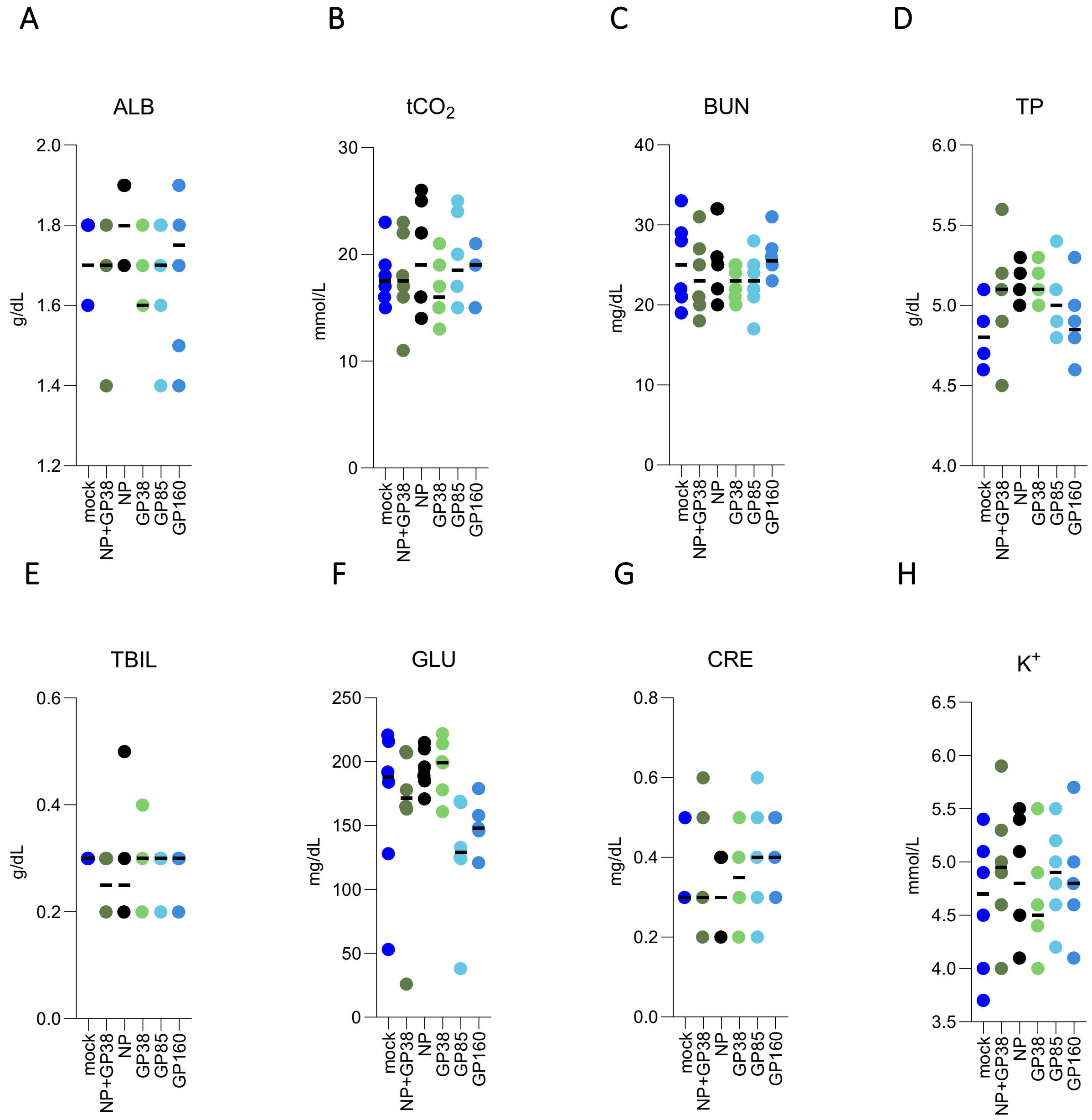

Supplementary Figure 2: Clinical chemistry analysis of blood samples four days after CCHFV challenge. Levels of albumin (ALB, g/dL) (A), total carbon dioxide content (tCO2, mmol/L) (B), blood urea nitrogen (BUN, mg/dL) (C), total protein (TP, g/dL) (D), total bilirubin (TBIL, g/dL) (E), glucose (GLU, mg/dL) (D), creatinine (CRE, mg/dL) (G), and potassium (K+, mmol/L) (H) were determined from whole blood within 1 h of collection. Horizontal line represents the median value for the group. Multiple comparisons were performed using a two-way ANOVA.

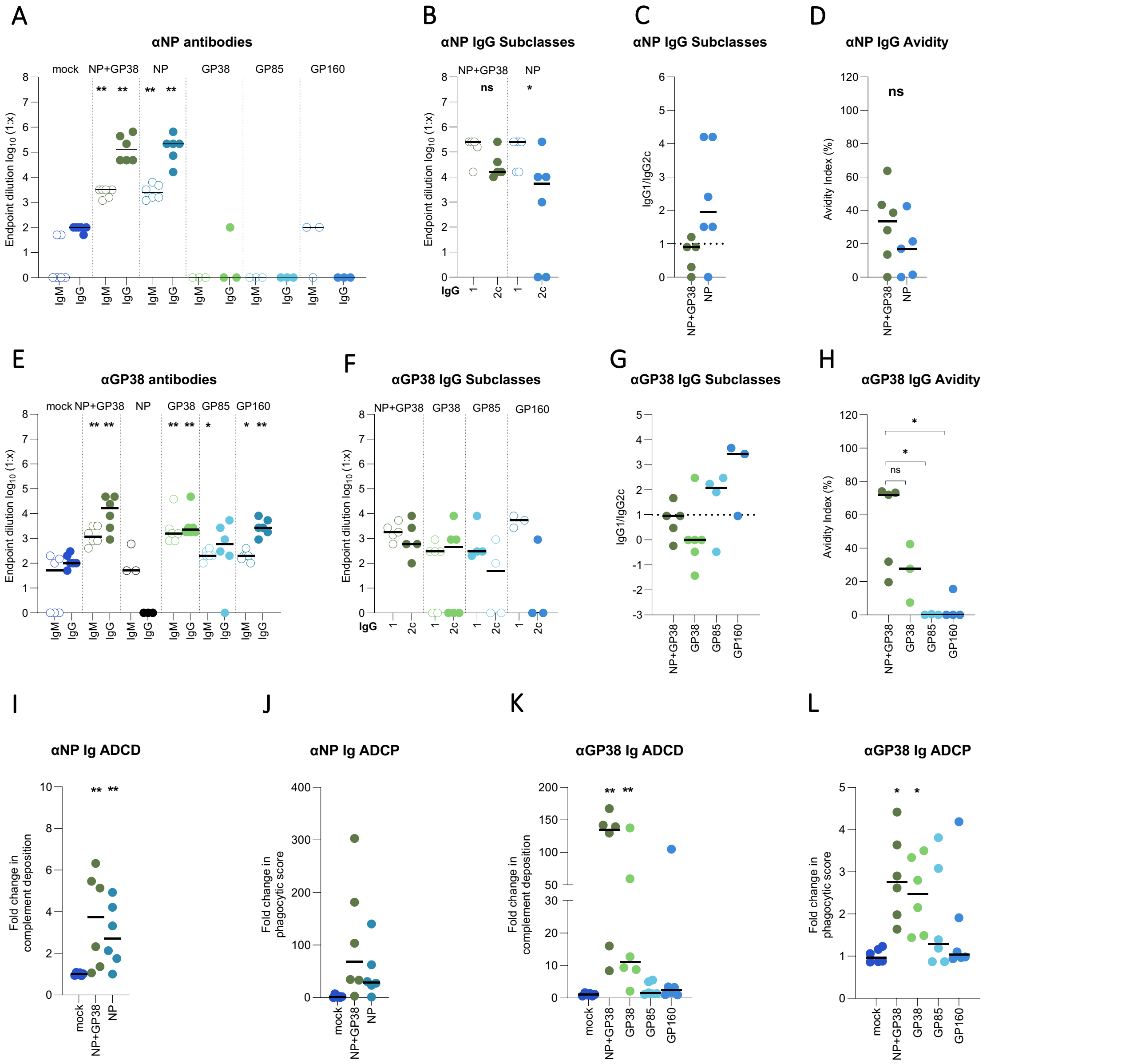

Supplementary Figure 3: Humoral immune responses four days after CCHFV challenge. Titers of anti-NP (A) and anti-GP38 (E) IgM and IgG, and subclasses of anti-NP (B) and anti-GP38 (F) IgG were given as endpoint titers. IgG1 to IgG2c ratios were determined by dividing the endpoint titers of IgG1 to IgG2 of individual animals and are shown in (C) for anti-NP antibodies and in (G) for anti-GP38 antibodies. The dotted horizontal line represents IgG1:IgG2c ratio = 1. The avidity indices of anti-NP (D) and anti-GP38 (H) antibodies four days after the challenge were determined. For the avidity of IgG antibodies, AUC of the 7-point dilutions of urea-treated and untreated samples were determined, and the avidity index was calculated as follows: (AUC of the urea-treated sample/AUC of untreated sample) × 100. The ADCD function of anti-NP (I) and anti-GP38 (K) antibodies represented as fold change in complement deposition over mock-vaccinated group. ADCP function of anti-NP (J) and anti-GP38 (L) antibodies represented as fold change in phagocytic score over mock vaccinated animals. Each dot on the graphs represents the mean value of the replicates from each animal and the horizontal line represents the median value for the group. All samples were tested in duplicate. Two-tailed nonparametric t-test and ordinary one-way ANOVA (* $p < 0.05$; ** $p < 0.001$; *** $p < 0.0003$) were used for statistical analyses when applicable. Statistical analyses were conducted comparing IgM and IgG titers from each vaccine group with those of mock-vaccinated animals. For IgG subclasses, statistical analyses were performed with IgG1 and IgG2 titers of individual vaccine groups. For IgG avidity, statistical analyses were performed with IgG titers of each vaccine group to compare differences in avidity in different vaccine groups.

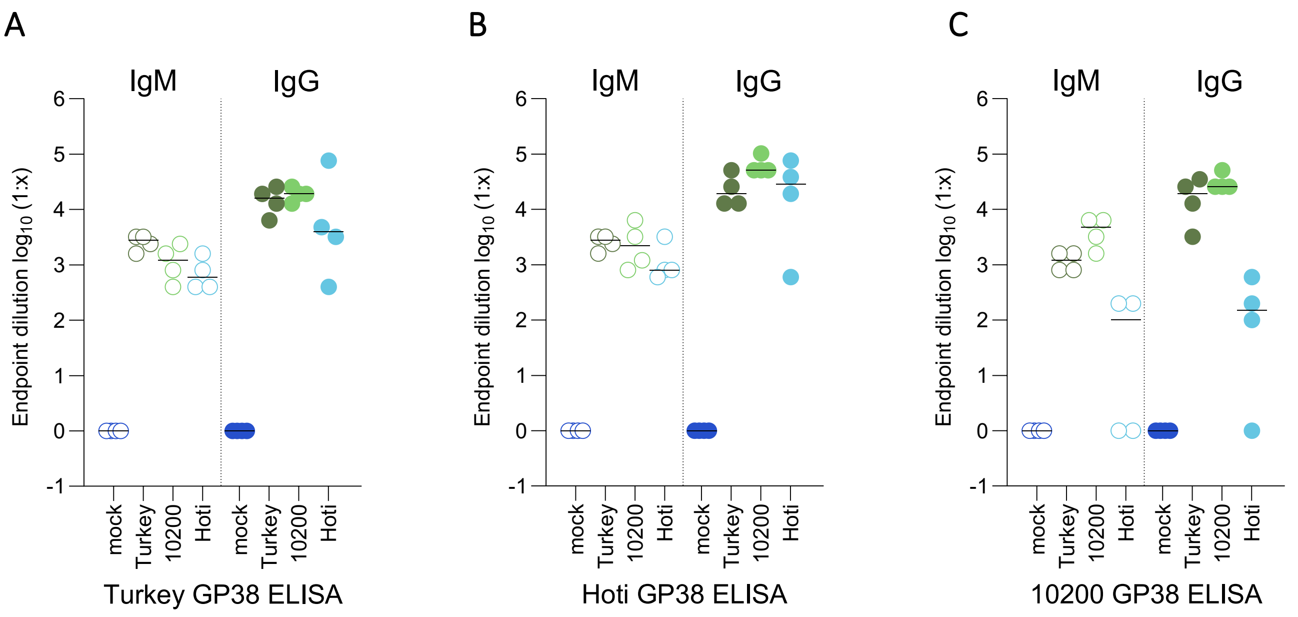

Supplementary Figure 4: Cross-reactivity of anti-GP38 antibodies. ELISAs were performed to detect differences in the binding of anti-GP38 antibodies to GP38 proteins from various CCHFV strains. (A) Plasma samples from animals infected with CCHFV Turkey or IbAr10200, as well as from Hoti GP38-vaccinated animals, were assayed to determine the binding of anti-GP38 IgM and IgG antibodies to GP38 proteins from different clades using recombinant GP38 of CCHFV Turkey (A), IbAr10200 (B), or Hoti (C) strains, and results were given as endpoint titers. Plasma samples from mock-vaccinated animals were used as controls. All samples were tested in duplicate. Each dot on the graphs represents the mean value of the replicates from each animal and the horizontal line represents the median value for the group. Two-tailed non-parametric t-tests and ordinary one-way ANOVA (*$p < 0.05$) were used for statistical analyses when applicable.

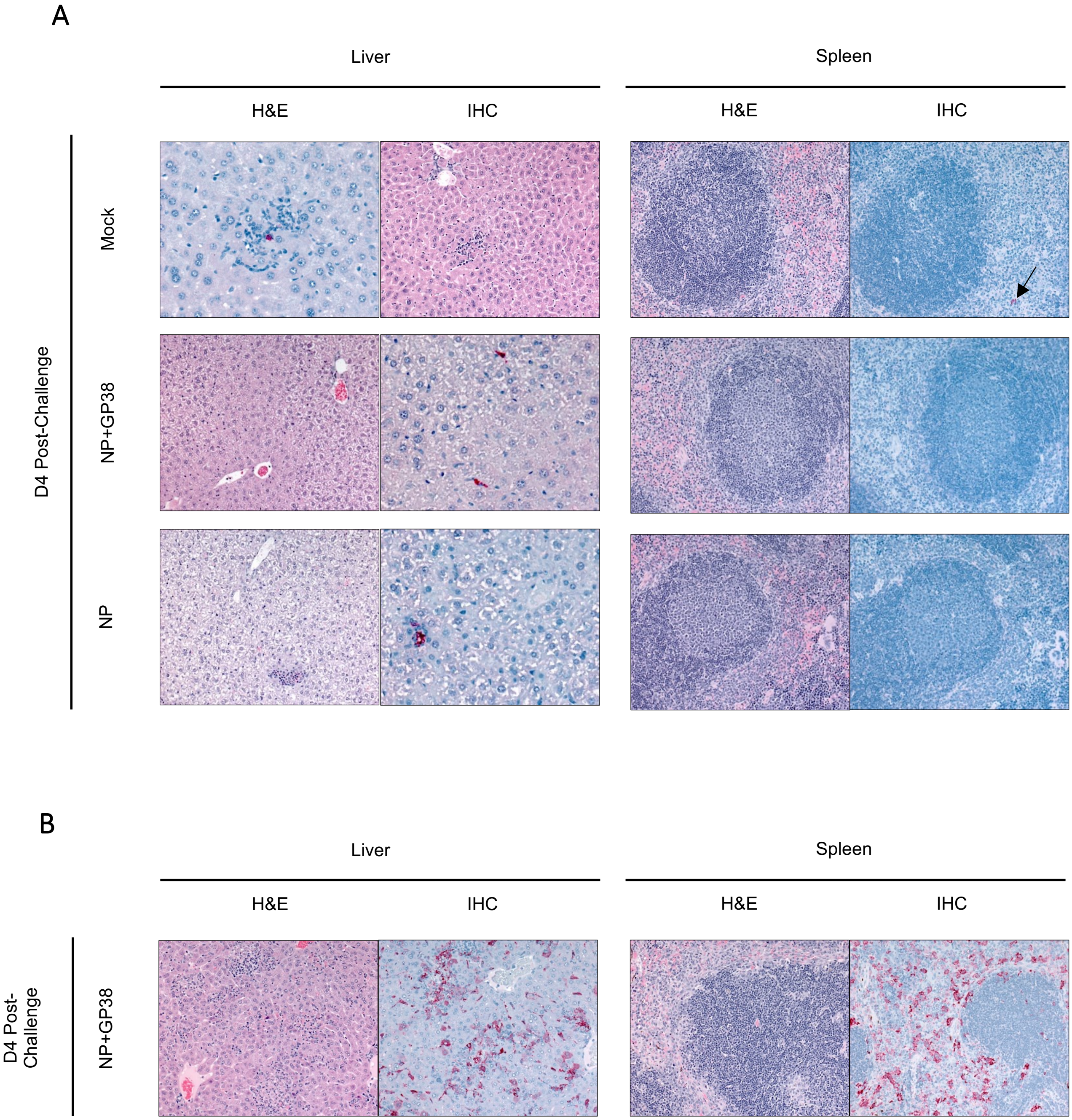

Supplementary Figure 5: Histopathological changes in liver and spleen of vaccine protected groups four days after CCHFV challenge. Groups of 6 animals from each vaccination group were euthanized 4 days after lethal challenge, and histopathology was conducted on liver and spleen samples from animals in the NP+GP38-, NP- and mock-vaccination groups were examined. (A) Representative images of histopathological findings and CCHF immunostaining in livers and spleens in mock-, NP+GP38- and NP- vaccinated animals. Most animals had similar findings in livers, with minimal inflammation and rare hepatocyte necrosis and IHC staining (red). Spleens from mock-vaccinated animals had few macrophages and neutrophils in the red pulp, and rare IHC staining (red; arrowhead), while spleens from both vaccinated groups showed splenic lymphoid reactivity and no IHC staining. (B) Two NP+GP38-vaccinated animals had more pathology and IHC staining, including one with widespread hepatocellular necrosis and inflammation, and prominent splenic change with increased macrophages.

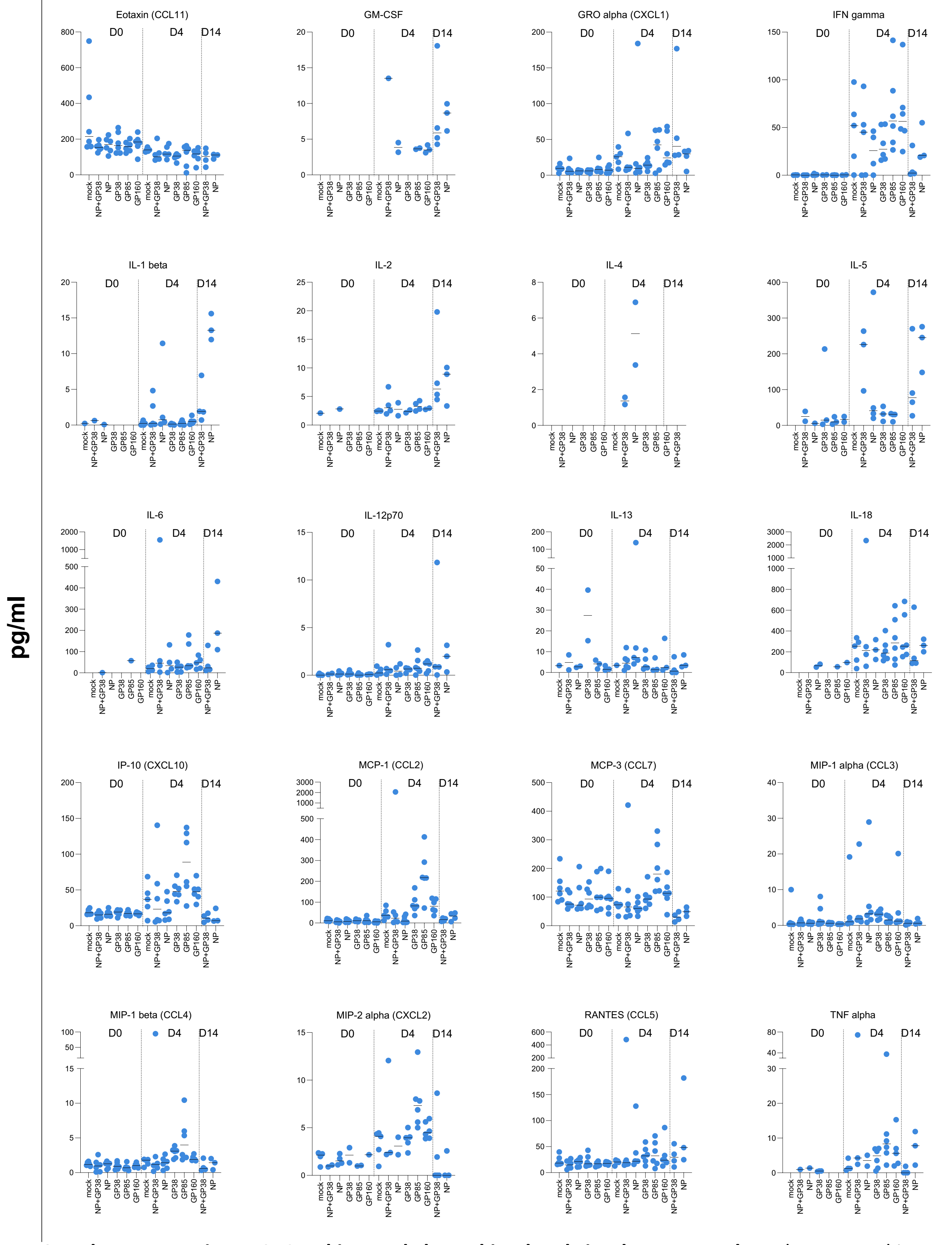

Supplementary Figure 6: Cytokine and chemokine levels in plasma samples. Plasma cytokine and chemokine levels were determined by using ProcartaPlex Mouse Th1/Th2 Chemokine panel. Results are represented in pg/mL. Horizontal line represents the median value for the group. Statistical analyses were performed by non-parametric one-tailed Mann-Whitney U-test to compare cytokine levels (*p < 0.1).

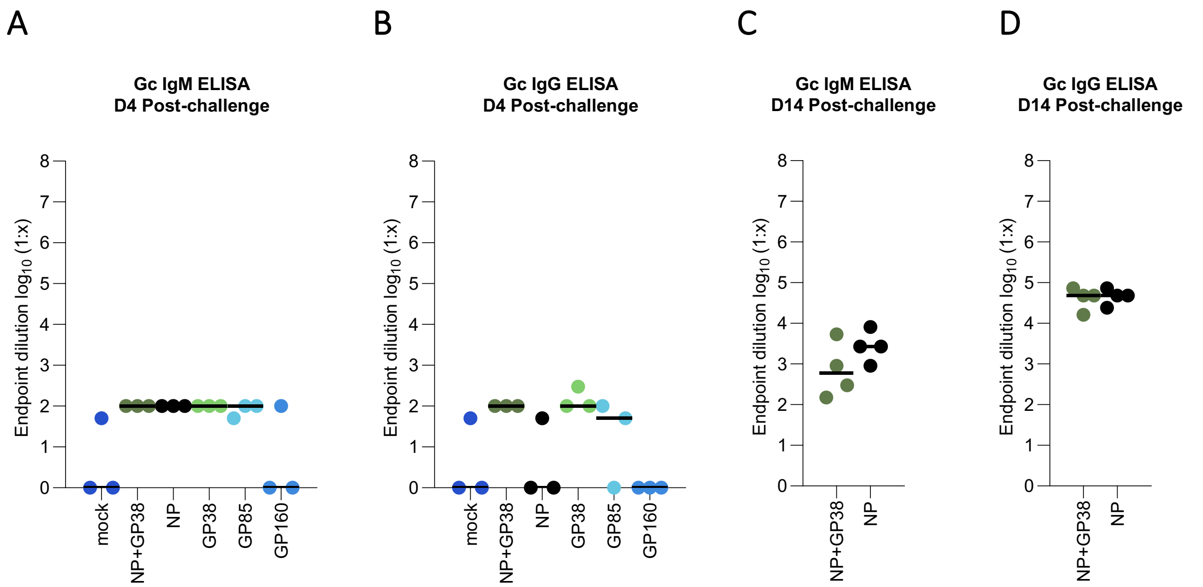

Supplementary Figure 7: Post-challenge antibody responses to viral antigen absent from the vaccines. Antibody responses to CCHFV Gc were investigated in plasma samples collected 4 and 14 dpc. Anti-Gc IgM and IgG are given as endpoint titers in (A) and (B) for samples collected on 4 dpc and in (C) and (D) for samples collected 14 dpc. All samples were tested in duplicate. Each dot on the graphs represents the mean value of the replicates from each animal and the horizontal line represents the median value for the group. Two-tailed non-parametric t-tests and ordinary one-way ANOVA (*p < 0.05) were used for statistical analyses when applicable.

Group	Animal ID	Weight loss from baseline (D0)		
		D6	D7	D8
Mock	3342-1-139	-6.5	-16.5	-18.4
	3342-1-140	-6.9	-14.7	-16.7
	3342-1-141	-13.6	-19.5	
	3342-1-142	-9.9	-14.2	
	3342-1-143	-12.4	-18.3	
	3342-1-144	-5.7	-15.3	-15.3
NP	3342-1-115	-1.9	-3.1	-1.1
	3342-1-116	-0.8	-5.5	-12.2
	3342-1-117	-8.8	-16.5	-22.1
	3342-1-118	0.0	-1.9	-4.3
	3342-1-119	-0.5	-11.2	-19.3
	3342-1-120	9.2	3.3	-3.8

Supplementary Table 1: Weight loss of animals from NP- and mock-vaccine groups to CCHFV challenge. Weight changes of animals represented as percent change from their baseline measurement at day 0 were given for 6, 7 and 8 days after the challenge with lethal dose of CCHFV. Greys boxes indicate no measurements available from animals that succumbed to infection 7 days after the challenge. Weight loss of animals in NP-vaccinated and mock-vaccinated groups 6 dpc (p = 0.015 and 7 dpc (p = 0.0173) were significantly different. Statistical analysis performed with two-tailed nonparametric t test.

Group	Animal ID	DPC	Liver - necrosis	Liver - inflammation	Liver IHC	Spleen - lymphoid	Spleen - inflammation	Spleen IHC
NP+GP38	3342-1-73	4	0	0	0	3	0	0
	3342-1-74*	4	4	4	4	1	3	4
	3342-1-75	4	0	0	1	3	0	0
	3342-1-76*	4	2	3	2	3	0	2
	3342-1-77	4	0	1	0	3	0	0
	3342-1-78	4	0	1	0	3	0	0
NP	3342-1-79	4	0	0	0	3	0	0
	3342-1-80	4	0	1	0	3	0	0
	3342-1-81	4	0	0	0	3	0	0
	3342-1-82	4	1	1	0	3	0	0
	3342-1-83	4	1	1	1	3	0	0
	3342-1-84	4	0	0	0	3	0	0
Mock	3342-1-103	4	0	1	0	1	1	0
	3342-1-104	4	0	0	1	0	1	1
	3342-1-105	4	1	2	1	1	0	1
	3342-1-106	4	1	1	0	0	0	0
	3342-1-107	4	2	3	3	1	2	3
	3342-1-108	4	1	0	1	1	1	0
NP+GP38	3342-1-109	14	1	1	0	2	0	0
	3342-1-110	7**	4	4	4	4	3	4
	3342-1-111	14	1	1	0	2	0	0
	3342-1-112	14	1	2	0	2	0	0
	3342-1-113	14	0	1	0	1	0	0
	3342-1-114	14	1	2	0	2	0	0
NP	3342-1-115	14	1	1	0	1	0	0
	3342-1-116	14	0	3	1	2	0	0
	3342-1-117	14	1	4	3	2	0	0
	3342-1-118	14	1	2	0	2	0	0
	3342-1-119	14	1	3	2	2	0	0
	3342-1-120	14	0	3	1	2	0	0
Mock	3342-1-139	8**	4	3	4	4	3	4
	3342-1-140	8**	4	3	4	4	2	4
	3342-1-141	7**	4	3	4	4	3	4
	3342-1-142	7**	4	3	4	4	2	4
	3342-1-143	7**	4	3	4	4	3	4
	3342-1-144	8**	4	3	4	4	3	4

Supplementary Table 2. Histopathology and immunohistochemistry scores for liver and spleen tissues from CCHFV-challenged mice. Livers were scored semi-quantitatively for hepatocellular necrosis and lobular inflammation, where 0 = not present, 1 = minimal, 2 = mild, 3 = moderate, 4 = severe. Spleens were scored semi-quantitatively for lymphoid changes, where 0 = not present, 1= mild reactive changes, 2 = moderate reactive changes, 3 = prominent reactive changes, 4 = lymphoid necrosis/apoptosis. Spleens were also scored for red pulp infiltration by neutrophils and macrophages, where 0 = not present, 1 = minimal, 2 = mild, 3 = moderate, 4 = severe. CCHFV immunostaining was also scored in liver and spleen, where 0 = no staining, 1 = rare, 2 = multifocal/mild, 3 = multifocal/moderate, 4 = extensive.
